# Negative Immune Checkpoint Inhibitors

**DOI:** 10.3390/pharmaceutics17060713

**Published:** 2025-05-28

**Authors:** Magda Drewniak-Świtalska, Paulina Fortuna, Małgorzata Krzystek-Korpacka

**Affiliations:** 1 Department of Biochemistry and Immunochemistry, Wroclaw Medical University, 50-368 Wroclaw, Poland; malgorzata.krzystek-korpacka@umw.edu.pl; 2 Omics Research Center, Wroclaw Medical University, 50-368 Wroclaw, Poland; paulina.fortuna@umw.edu.pl

**Keywords:** immune checkpoint, small-molecule inhibitors, peptide inhibitors, PROTAC

## Abstract

Checkpoint inhibitors are a modern therapeutic approach for treating various types of cancer, metabolic diseases, and chronic infections. The main goal of this therapy is to specifically unlock the immune system, allowing it to recognize and eliminate cancer cells or pathogens, primarily through the activation of T lymphocytes. Monoclonal antibodies used in the treatment of various cancers, such as pembrolizumab (Keytruda), nivolumab (Opdivo), and ipilimumab (Yervoy), carry several limitations, primarily due to their large molecular size. The main challenges include limited tissue penetration, long half-life in the body, and the risk of autoimmune responses. Compared to antibodies, small-molecule and peptide inhibitors offer significant advantages related to their molecular structure. These drugs demonstrate a better ability to penetrate hard-to-reach areas, such as the tumor microenvironments, can be administered orally, and often show lower immunogenicity. A new generation of drugs is PROTACs, which combine the ability to direct proteins to degradation with the action of checkpoint inhibitors, contributing to the elimination of proteins responsible for suppressing the immune response. This publication describes small-molecule inhibitors, peptide inhibitors, and PROTAC molecules targeting negative immune checkpoints—CTLA-4, PD-1, VISTA, TIM-3, BTLA-4, LAG-3, and TIGIT.

## 1. Introduction

The main objective of immune checkpoint inhibitors (ICIs) is to restore the functionality of immune cells, particularly T lymphocytes, by binding to specific molecular targets [1]. T cells are responsible for maintaining immune homeostasis in the body and can penetrate the tumor microenvironment (TME) [2]. Modulating the function of these cells and activating them may be a potential therapeutic approach for treating various immune disorders and cancers [1,2]. In 2000, few scientists believed in the success of cancer immunotherapy used in research and later in clinical treatment [3]. A significant contribution to the development of this field was made by two scientists, James P. Allison and Tasuku Honjo, who were recognized by the Nobel Prize Committee in 2018 for their discovery of cancer therapy through the inhibition of negative immune regulation. Their work focused on discovering the first negative immune checkpoints (ICs)—PD-1 and CTLA-4 [4]. Since then, several other negative receptors have been identified: VISTA can act as both a ligand and a receptor [5]; BTLA is involved in a network of interactions that both inhibit and stimulate T cell activity [6]; LAG-3 functions through direct binding to MHC class II; TIM-3 has numerous ligands, including galectin-9, which is not membrane-bound; and TIGIT blocks the activating signal of CD226 [7]. In addition to negative ICs, positive ICs have also been identified, which play a role in enhancing the activity of immune cells. For instance, SIRP-α functions as a regulator of phagocytosis by macrophages and neutrophils [8], while OX40 may contribute to the strengthening of immunological memory [9].

The first ICIs approved by the FDA were Ipilimumab, a human monoclonal anti-CTLA-4 antibody, and Pembrolizumab and Nivolumab targeting the PD-1/PD-L1 interaction, all initially used in the treatment of melanoma [10,11]. The therapeutic potential of these mAbs was expanded to other cancers, such as non-small-cell lung cancer (NSCLC), renal cell carcinoma, urothelial carcinoma, and Hodgkin lymphoma [12]. However, using antibodies in monotherapy is not practical in all cases. Therefore, combination therapy was introduced, aiming to simultaneously block multiple IC pathways, such as Nivolumab (PD-1/PD-L1) plus Relatlimab (LAG-3) [13]. The introduction of immune checkpoint therapy (ICT) revolutionized the approach to cancer treatment, becoming, alongside chemotherapy, radiotherapy, and surgery, a cornerstone of therapy [10].

Despite the undeniable advantages of monoclonal antibodies (mAb) used in ICT, as molecules introduced into the living organism, they carry several significant obstacles. The mAbs are characterized by poor pharmacokinetic (PK) profiles. Pharmacokinetics is the study and characterization of the time course of drug absorption, distribution, metabolism, and excretion (ADME) [14]. Starting with the route of administration, mAbs cannot be administered orally due to degradation in the gastrointestinal tract. Intravenous or subcutaneous administration is therefore necessary, which significantly reduces patient convenience. Due to their large molecular size, mAbs have limited distribution throughout the body, and penetration into tissues such as solid tumors is often restricted. This can lead to a delay in the onset of therapeutic effects. The long half-life of mAbs can be both an advantage—allowing for less frequent dosing—and a drawback. Prolonged exposure to foreign antibodies can trigger immune responses, potentially leading to various adverse effects [14,15,16,17]. Patients treated with CTLA-4 inhibitors report side effects in up to 90% of cases, while those treated with PD-1/PD-L1 inhibitors report them in approximately 70% of cases. Immune-related adverse events (irAEs) can occur across various systems, including gastrointestinal, hematologic, endocrine, respiratory, neuromuscular, cardiovascular, dermatologic, and urinary systems [18]. Increased immunogenicity can also reduce therapeutic efficacy by accelerating the clearance of mAbs from the body or triggering allergic reactions. In addition to ADME-related challenges, mAbs are complex molecules whose synthesis is time-consuming, technically demanding, and costly [19,20,21]. The development of therapeutic mAbs requires careful selection and immunization of appropriate cell lines, large-scale cell culture, the introduction of post-translational modifications such as glycosylation, and subsequent isolation and purification of the antibody product [22].

Unsurprisingly, alternative strategies acting as ICIs are being actively explored. In contrast to mAbs, ICT utilizing small molecules and peptides offers numerous advantages. Firstly, the large-scale synthesis of chemical compounds is significantly more cost-effective and time-efficient compared to the complex and resource-intensive production of mAbs with similar therapeutic potential. Furthermore, small molecules and peptides exhibit more favorable PK profiles. Their relatively short half-lives reduce systemic accumulation risk facilitating more predictable pharmacodynamics, easier dose optimization, and improved toxicity management. These compounds also exhibit significantly lower immunogenicity than mAbs, making them particularly suitable for long-term or chronic treatment regimens, where sustained safety and predictable immune responses are essential. Another undeniable advantage is their superior oral bioavailability greatly enhancing patient comfort and compliance. Oral administration substantially reduces the logistical and financial burden of parenteral delivery methods such as intravenous or subcutaneous injections. In addition, the smaller molecular size of these compounds confers multiple pharmacological benefits. They demonstrate greater ability to penetrate tumor tissues—an essential feature in the treatment of solid tumors. Moreover, their capacity to traverse cellular membranes enables them to access intracellular targets, which is a therapeutic domain largely inaccessible to larger biologics such as mAbs due to their size and hydrophilic nature [21,23,24].

PROTACs (PROteolysis-TArgeting Chimeras) represent an innovative drug design technology aimed at targeted protein degradation, with rapidly growing applications in the treatment of cancer and other protein-driven diseases [25]. These heterobifunctional small molecules comprise three key elements: a ligand that binds the target protein, a ligand that recruits an E3 ubiquitin ligase, and a chemical linker that connects the two moieties into a single functional entity. The linker’s length and flexibility are optimized to bring the target protein and the E3 ligase into close spatial proximity. This enables the PROTAC molecule to act as a “molecular bridge”, facilitating the transfer of ubiquitin from the E3 ligase to the target protein, which is subsequently directed to degradation by the 26S proteasome [26,27,28]. Unlike traditional inhibitors that act by blocking enzymatic activity or protein–protein interactions, PROTACs lead to the complete removal of the target protein from the cell. Therefore, they do not function as classical point inhibitors ICIs but rather exert their therapeutic effects indirectly by eliminating entire receptors, ligands, or other regulators of immune or signaling pathways. PROTACs operate catalytically—once they initiate degradation, they are not consumed. This allows them to be reused multiple times within the cell until they are naturally metabolized or eliminated from the body. This unique property enables the use of lower therapeutic doses, potentially reducing toxicity and improving the overall safety profile of treatment [26,27].

The first PROTAC molecule to enter Phase II clinical trials was **ARV-110**, which was developed by Arvinas. Its mechanism of action is based on the targeted degradation of the androgen receptor (AR) via recruitment of the E3 ubiquitin ligase cereblon (CRBN). ARV-110 was designed for the treatment of metastatic castration-resistant prostate cancer (mCRPC). Preliminary clinical data indicate antitumor activity in patients with AR mutations that confer resistance to conventional hormonal therapies and a favorable safety profile [27,29]. The most clinically advanced PROTAC is **ARV-471**, which was co-developed by Arvinas and Pfizer. This compound selectively degrades the estrogen receptor alpha (ERα), utilizing CRBN as the recruiting E3 ligase. ARV-471 is being developed for the treatment of ER-positive, HER2-negative breast cancer. Earlier-phase studies demonstrated efficient ER degradation, promising disease control rates, and tolerability. It is now in Phase III clinical trials [29].

**Figure 1:**
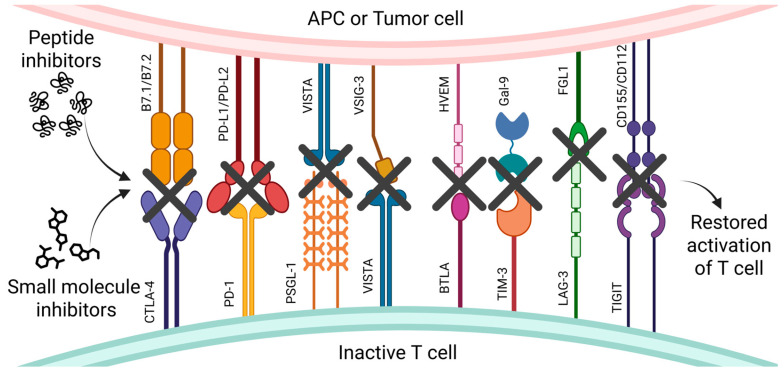
The effect of small-molecule and peptide inhibitors on negative immune checkpoint receptors and their ligands on inactive T cells. The figure shows the schematic interactions between negative ICs on inactive T cells and their ligands expressed on antigen-presenting cells (APCs) or tumor cells. These interactions—PD-1/PD-L1 or PD-L2, CTLA-4/B7.1 or B7.2, PSGL-1/VISTA and VISTA/VSIG-3, BTLA/HVEM, TIM-3/Gal-9, LAG-3/FGL1, and TIGIT/CD155 or CD112—suppress T cell activity and enable immune evasion by tumor cells. The graphic highlights the blockade of these interactions using small-molecule and peptide inhibitors (black cross). The ligands and receptors are depicted as colorful, membrane-bound structures forming inhibitory signaling pairs between cells; their shapes approximately reflect the actual molecular architecture of these proteins. Blocking these pathways leads to T cell reactivation, restoring their ability to recognize and eliminate tumor cells. Created with BioRender.com.

In this review, we present the latest information on small molecules, peptides, and PROTACs as inhibitors of CTLA-4, PD-1, VISTA, TIM-3, BTLA-4, LAG-3, and TIGIT, along with a brief description of their structures, ligands, and mechanisms of action in both healthy and diseased organisms (Figure 1).

## 2. CTLA-4

### 2.1. CTLA-4 Structure

Cytotoxic T-lymphocyte-associated molecule 4 (CTLA-4), also known as CD152, is a type 1 transmembrane glycoprotein that belongs to the CD28 immunoglobulin subfamily expressed on the surface of activated T cells [30,31] that is capable of interacting with other B7-family proteins members—B7-1 (CD80) [32] and B7-2 (CD86) [33]. CTLA-4 is a key regulator of T cell homeostasis and autoimmunity [34].

The structure of CTLA-4 consists of 233 amino acids, forming a V-type domain surrounded by two hydrophobic regions, one of which is anchored in the membrane [30]. The region interacting with the B7-1/B7-2 proteins is formed by two layers of five strands in β-sandwiches [32,33]. A complementary determining region 3 (CDR3)-like segment is formed between strands F and G, with the sequence Met-Tyr-Pro-Pro-Pro-Tyr-Tyr, which is capable of forming strong interactions through hydrogen bonds and van der Waals contact with B7-2 [32,33]. CTLA-4 is expressed on T cells as a dimer, which forms primarily due to hydrophobic interactions between strands A and G, as well as an interchain disulfide bond between two Cys 122 residues [32]. This dimerization does not change the interaction surface with the B7-2 protein [33]. The cytoplasmic domain, composed of only 37 amino acids, contains the YVKM sequence, with which it interacts with the lipid kinase phosphatidylinositol 3-kinase (PI3K) [35], the phosphatase SHP-2, which is suspected to be a critical transducer of negative signaling from CTLA-4 [36], PP2A [37], and the clathrin adaptor proteins AP-1 and AP-2 [38,39].

In the PDB database, you can find as many as 20 different crystal structures of the CTLA-4 protein (in full length as well as in fragments—human or mouse sequences) in various complexes—bound with the B7-1 ligand [32] and B7-2 [33], with antibodies [11,40,41,42,43,44,45,46,47], with phosphoinositide 3-kinase SH2 domains [48], with the AP50 subunit of AP-2 [49], and with miniproteins [50].

### 2.2. CTLA-4’s Expression in Health and Disease

Apart from T cells and regulatory T cells (Treg), CTLA-4 is expressed in different cells, including B cells [51,52,53], monocytes [54], granulocytes [55], and placental fibroblasts [56], but not in non-hematopoietic cells [39].

Transcription factors positively correlated with the expression of CTLA-4 protein include Nuclear Factor of Activated T Cells (NF-AT), NF-κB, and AP-1 [57]. Inhibition of these factors reduced CTLA-4 transcription [58]. Additionally, the expression of CTLA-4 is upregulated by cAMP and Ca^2+^ [59]. 

The mechanism explaining the inhibitory effect of CTLA-4 on T cell proliferation and activation is not fully understood [60]. CTLA-4 enhances cell motility by limiting stable interactions between T cells and antigen-presenting cells (APCs) [38]. Several options for this type of regulation have been reported. CTLA-4 competes with the CD28 receptor expressed on naive T cells, showing a higher affinity for the same B7 proteins: B7-1 (CD80) and B7-2 (CD86) expressed on APCs [39]. Despite quaternary structure similarities (30% identity [31]), CD28 and CTLA-4 exhibit different sequences in the dimerization region, which may explain the lack of formation of CTLA-4/CD28 heterodimers [61]. Little resemblance could indicate a distinct effect of these two receptors; the CD28 receptor is responsible for activation, while CTLA-4 prevents activation of naive T cells [34]. CTLA-4 can also inhibit the expression of lipid rafts or be present within the rafts themselves, reducing the number of T cell receptors important for T cell signaling [62]. Additionally, upon binding the B7-1 or B7-2 ligand, the cytoplasmic domain of the CTLA-4 receptor undergoes phosphorylation. The involved phosphatases, SHP-76 and protein phosphatase 2A (PP2A), inhibit the intracellular signaling of the T cell receptor (TCR) and CD28 receptors, thereby reducing T cell proliferation and interleukin-2 (IL-2) production [63,64].

The essential role of CTLA-4 has been associated with several autoimmune diseases, such as Hashimoto’s disease and Graves’ disease [65], autoimmune hemolytic anemia [66], hypogammaglobulinemia [67], and B cell lymphopenia [68,69].

CTLA-4 is also associated with cancer cells, although the exact mechanism of this regulation is not fully understood [70]. Studies on murine leukemia cells confirm the role of CTLA-4 in preventing cancer cells from escaping the immune response [71]. Cancer cells, as abnormally growing cells with point mutations in DNA, can be detected and attacked by the immune system as foreign cells [34]. Several mechanisms have been proposed to justify the therapeutic inhibition of CTLA-4 interactions with its receptors and other factors within TME. For instance, CTLA-4 may interact with SHP2 not only via its binding motif but also through direct contact with tyrosine residues, leading to suppression of the TCRζ signaling pathway. Additionally, CTLA-4 can disrupt the formation of ZAP70 clusters on the surface of T cells in the TME, which are essential for effective T cell activation signaling [72]. Blocking CTLA-4, particularly on Tregs, is believed to enhance antitumor immune responses by promoting the infiltration of cytotoxic T cells and simultaneously limiting the recruitment of Tregs into the TME [73]. 

Due to the likely mechanism of action (anti-CTLA-4), immunotherapy seems promising for treating various types of malignant cancers [36,74,75]. So far, the literature has described antibodies that demonstrated therapeutic properties against, among others, prostate cancer [64,76,77], melanoma [78,79,80], lung cancer [81,82], and ovarian cancer [83,84].

### 2.3. CTLA-4 as a Receptor

As mentioned earlier, CTLA-4 is a receptor that interacts with two proteins, B7-1 and B7-2. A vital difference affecting these interactions is the structure of these proteins. B7-1 (CD80) is a non-covalent homodimer with two CTLA-4 binding sites (low and high avidity), while B7-2 (CD86) is a monomer with low avidity [62,85]. These different mechanisms involving the B7-1 and B7-2 proteins reduce their availability for CD28, a monomer, and decrease the number of activated T cells [39]. CD28 binds to B7 ligands with a 50- to 200-fold lower affinity than CTLA-4 [64].

Differences in the sequences of CTLA-4 and CD28 in the receptor-binding region may have several implications [33]. The interaction regions between B7-1/B7-2 and CTLA-4 form a β-sandwich structure, with its surface creating a shallow, concave surface due to the natural twist of the structure [33]. It is worth noting that binding to B7-2 does not induce any significant conformational changes in either the monomeric or dimeric forms of CTLA-4 [33]. Similarly, in the case of CTLA-4 and B7-1 monomers, the conformational changes upon binding are minimal and are localized solely in the interaction area [32]. An important observation is that disulfide bonds enforce the conformation of the CTLA-4 dimer so that homodimers of B7-2 interact distally with the CTLA-4 dimer, promoting the binding of two independent B7-2 complexes [33]. This is in contrast to the B7-1 proteins, which interact orthogonally [32] and do not form stable homodimers [86].

Thus, these structural and conformational differences arising from the distinct sequences of CTLA-4 and CD28 may influence the way these receptors interact with their ligands and the formation of complexes, potentially affecting their functional outcomes in immune signaling.

### 2.4. CTLA-4’s Inhibitors

Due to the lack of a pocket binding on the surface of the CTLA-4 receptor, obtaining inhibitors that interfere with ligand interactions may be challenging. Only a few peptides and small molecules that inhibit the interaction of CTLA-4 with the B7-1 or B7-2 proteins have been identified, thereby inhibiting T cell deactivation.

#### 2.4.1. Peptides

**Figure 2:**
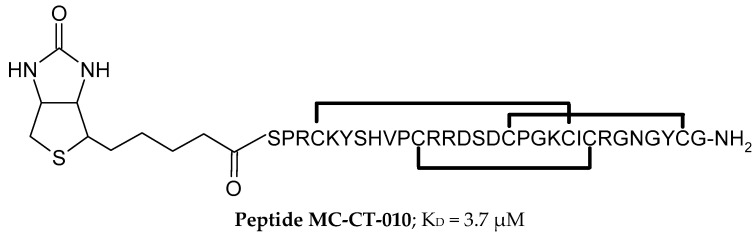
Structures of CTLA-4 peptide inhibitors **MC-CT-010**.

The first described inhibitors of the CTLA-4/B7-1 and CTLA-4/B7-2 interactions were peptides whose sequences were based on phage display technology [87]. Two motifs, **peptide F2** (GFVCSGIFAVGVGRCGAAGAET) and **peptide F6** (APGVRLGCAVLGRYCGAAGAET), were selected, which form a tertiary structure with an antiparallel β sheet stabilized by internal disulfide bridges, forcing the formation of an internal loop [87]. Another example of peptides **MC-CT-010** (Figure 2) that inhibit the CTLA-4/B7 interaction, which also has stabilizing internal disulfide bridges, are derivatives of the cysteine-knot protease inhibitor (McoTI-II) obtained through the strategy of high-throughput screening (HTS) of combinatorial libraries [88]. Additionally, the obtained peptides were subjected to oligomerization using a biotin/streptavidin system to generate di-, tetra-, and heptameric forms of the ligand (similar to the B7-1 protein) and thereby increase affinity for CTLA-4 [88]. The K_D_ value of the CTLA-4/MC-CT-010 interaction, determined using ELISA, was 3.7 μM [88].

The helix–loop–helix (HLH) peptide is an example of a peptide with a tertiary structure that inhibits the CTLA-4/B7 interaction [89]. Based on yeast-displayed libraries of HLH peptides and their interaction with CTLA-4, several peptide sequences were proposed, among which **peptide Y-2** (CAWGLAILEGELAWLEGGGGGGGQLAD LKRQLAWLKQAC) exhibited an affinity for CTLA-4 with a K_D_ value of 6.4 ± 0.15 μM in SPR experiments [89]. Based on the peptide Y-2 sequence, another series of synthetic peptides was proposed, among which **peptide ERY2-4** (cyc(CAWGQAILEGELAWLEGGGGGAGQLADLKRQLAWWKQAC)) exhibited a K_D_ value of 196.8 ± 2.3 nM in SPR experiments and specifically inhibited the CTLA-4/B7-1 interaction without affecting the CD28/B7-1 interaction [89].

Based on the crystal structure of the CTLA-4/B7-2 complex and computational techniques (FlexPepDock as a module of Rosetta and molecular mechanics generalized Born surface area MM-GBSA), a cyclic **peptide 16** (EIDTVLTPTGWVAKRYS) with a β-hairpin structure and a PTG turn was proposed, which binds to the CTLA-4 receptor at the same site as the B7-2 protein [90]. Unfortunately, this peptide’s experimentally measured dissociation constant (biolayer interferometry) was relatively low, with a K_D_ = 31 ± 4 μM [90].

An additional example of a peptide interacting with the CTLA-4 protein is the PRS-010#003 (PDB 3BX7), consisting of 178 amino acid residues, whose sequence and structure are based on lipocalin [91]. Lipocalins are proteins with a conserved structure based on an eight-stranded antiparallel β-barrel and a helix on the side, with a hydrophobic core and four loops [92], whose structure is similar to that of B7-family proteins. The dissociation constant for this peptide was K_D_ = 9.00 ± 0.02 nM, which was determined by Biacore analysis [91].

Phage display peptide library screening also led to the identification of the **peptide LC4** (WGHSHFSHWKGR), which exhibited affinity (K_D_ = 6.86 ± 2.63 μM) for CTLA-4, inhibiting the CTLA-4/B7 interaction [93]. A linker with the sequence PLGLAG was added to the peptide, enabling conjugation to RGD—a sequence that shows high affinity for the integrin ανβ3 and targets colon tumor tissue [93].

Another approach to designing peptides that inhibit the CTLA-4/B7 interaction involved creating CTLA-4 analogs with sequences based on regions critical for this interaction: CDR1 and CDR3. This led to the proposal of a peptide with the **peptide EL16** with sequence AVCELMYPPPYYLCIS [94]. Since the FN3 protein (human type III fibronectin; PDB: 1TTF) shows structural similarity to the CTLA-4 protein (PDB: 1I8L) in regions complementary to CDR1 (8 amino acid residues) and CDR3 (10 amino acid residues), sequences derived from CTLA-4 were inserted, resulting in the creation of a new protein, CFN13, which exhibited properties that block the CTLA-4/B7-1 interaction [94].

#### 2.4.2. Small Molecules

**Figure 3:**
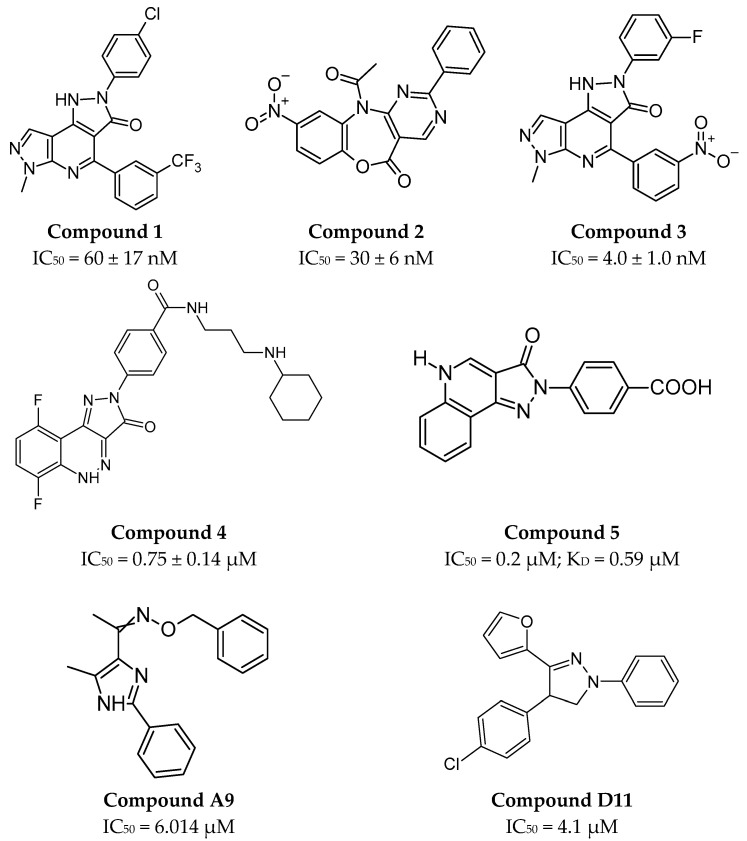
Structures of CTLA-4 small-molecule inhibitors.

Different approaches to inhibiting the CTLA-4/B7 interaction involve small molecules interacting with the B7-1 protein. One of the first described small molecules interacting with B7-1 included three derivatives of dipyrazolopyridinone compounds (**compound 1**, **2**, and **3**, Figure 3), which, despite having different molecular structures, show a direct overlap of binding epitopes on the B7-1 protein [95].

After the publication of studies on compound **1** [95], a series of different derivatives inhibiting the CTLA-4/B7-1 interaction was proposed. Based on a scintillation proximity assay (SPA), time-resolved fluorescence resonance energy transfer (TR-FRET) assay, and SPR experiments, a lead structure consisting of pyrazoloquinolinone was proposed, along with more potent analogs (**compound 4** with the best IC_50_ parameter; see Figure 3) [96]. In addition to the cell-based homogeneous SPA method, distance-related energy transfer was used to discriminate between bound and unbound ligands in a free-cell format [97]. Optimization, including the design and synthesis of six structural analogs of compounds **1**–**6** [96], led to the identification of several B7-1 inhibitors (**compound 5** with the best IC_50_ parameter; see Figure 3), which should be further improved when considering their development as marketable drugs [97]. A comprehensive series of derivatives (several dozen compounds) was also obtained through a rational approach involving the substitution of various functional groups (e.g., phenyl, chlorinated derivatives, aryl, etc.) at the interaction sites with B7-1. This was achieved by dividing compound 1 into northern and southern sections [98]. However, as a result of further studies, it was found that compound **1** (RhuDex^TM^, AV1142742) blocks the CD28/B7-1 interaction, meaning that such a series of compounds cannot be considered as small molecules directly blocking the CTLA-4/B7-1 interaction [99].

AI has also been used to design small molecules interacting with the CTLA-4 receptor entirely in silico [100]. Five compounds were proposed, two of which, **compound A9** and **compound D11** (Figure 3), triggered an immune response that inhibited tumor growth at concentrations of 25 mg/kg [100]. However, the mechanism by which this occurs requires further investigation and characterization due to the involvement of macrophage cells as well [100]. From the perspective of AI development and its integration with systems like AlphaFold, it may become possible to predict the three-dimensional structures of proteins based on their amino acid sequences [100].

## 3. PD-1

### 3.1. PD-1 Structure

PD-1 (Programmed death-1), also known as CD279, is a type I transmembrane glycoprotein belonging to the CD28 immunoglobulin subfamily [101]. PD-1 is expressed on the surface of activated T lymphocytes and B lymphocytes [102], as well as on myeloid cells [103]. It consists of 288 amino acid residues, which form a characteristic extracellular immunoglobulin variable-type (IgV) domain, a transmembrane region, and a cytoplasmic tail [104,105]. The intracellular domain of PD-1 contains two essential motifs: the immunoreceptor tyrosine-based inhibitory motif (ITIM) [106] and the immunoreceptor tyrosine-based switch motif (ITSM), which engages the intracellular phosphatase SHP2 (SRC homology 2 (SH2)-domain-containing protein tyrosine phosphatase 2) [107]. The function of SHP2 is dephosphorylate, and it deactivates downstream signal transducers, including reducing the secretion of IL-2 [108]. These intracellular interactions are not fully understood [101].

The extracellular domain contains two hydrophobic regions arranged into a β-sheet structure with CC’FG on the front face and AA’BDE strands on other sites [105]. Two cysteine residues in the β-sheet structure (Cys54 and Cys123) form a disulfide bond similar to that found in the immunoglobulin domain of the V-set sequences [104]. However, the second pair of such residues, which would form homodimeric structures as in other members of this subfamily [32], is absent. Therefore, PD-1 exists in a monomeric form both in solution and on the cell surface [102,105]. Despite the presence of the CDR3 loop, these residues, unlike other members of the CD28 subfamily, are not critical for interactions with ligands [105]. Instead, the residues located on the receptor surface in the CC’FG strands [105] are critical, forming a binding site for the two ligands, PD-L1 (B7-H1) [103] and PD-L2 (B7-DC) [109,110]. The conformational structure of PD-1 does not undergo a significant change in solution upon interaction with the PD-L1 ligand [111] and a much smaller change upon binding PD-L2, which may be insufficient for direct signal transduction across the plasma membrane [109].

Despite a 23% sequence similarity to other members of the CD28 immunoglobulin subfamily [103], all these features position PD-1 as the most unique and, therefore, the most studied checkpoint of immune response regulation [105].

### 3.2. PD-1’s Expression in Health and Disease

Due to the type of cells on which the PD-1 receptor is expressed, it is primarily involved in processes that deliver signals regulating the balance between T cell activation in response to foreign and self-antigens, i.e., tolerance and immunopathology [112] related to the immune system, including immunity against infections, transplant rejection, allergies, cancer [113], and autoimmunity [103].

In a healthy body, naive T cells are activated through the interaction of the MHC complex with TCR and a co-stimulatory signal delivered to T cells by APCs, thus increasing the number of activated T cells and inhibiting T cell proliferation and cytokine secretion [112,114]. This results from the interaction of the PD-1 cytoplasmic region with SHP2 and the inhibition of phosphorylation of tyrosines in various signaling molecules [103]. Targeting and increasing the number of T cells resulting from blocking the PD-1/PD-L interaction could be a primary mechanism in immunotherapy for cancer [115], but in therapeutic strategies, energy deficiencies and translational issues in exhausted T cells may also be significant [116]. Further research on the impact of PD-1 on T cell survival, which is dependent on CD28, IL-2 [117], and Bcl-xL, is needed [112]. As a negative receptor, PD-1 can regulate effector T cells and suppress the early activation of naive T cells [112]. PD-1, a checkpoint in activated T cells, acts later in the immune response than CTLA-4, which may explain the milder course of diseases caused by the proliferation of T cells lacking PD-1 compared to CTLA-4 [34,103]. Abnormalities in the expression of PD-1 protein and its ligands, PD-L, lead to pathological states in the body, such as chronic, metabolic, and oncological diseases [118].

Microorganisms causing chronic infections may exploit the PD-1/PD-L pathway to evade the immune response of the attacked organism [112]. In the case of acute illnesses and vaccinations, effector T cells develop and differentiate into memory T cells [115]. In chronic infections and cancer, antigen stimulation occurs continuously, decreasing in the pool of effector T cells characterized by high PD-1 receptor expression, followed by T cell exhaustion [114,115,116,119]. T cell exhaustion concerns chronic diseases such as lymphocytic choriomeningitis virus (LCMV) [114,117,120], HIV [121,122,123], hepatitis B (HBV) [124,125,126], and C (HCV) [127,128,129], as well as metabolic diseases such as type 1 diabetes [130], Metabolic Dysfunction-Associated Steatotic Liver (MASL) [131], and rheumatoid arthritis [132,133]. Cancers in which pathological changes in PD1/PD-L expression have been identified include melanoma [134,135], chronic myelogenous leukemia [136,137] and lymphocytic leukemia, ovarian cancer [138,139], NSCLC [140,141], and Hodgkin’s lymphoma [142].

Recent studies indicate that PD-1 is not exclusively expressed on immune cells but may also be present as an intracellular variant (iPD-1) within cancer cells, where it plays a significant role in malignant progression [143]. The expression of PD-1 on non-immune cell types may partly account for the heterogeneous therapeutic responses observed with anti-PD-1 mAb treatments across various tumor types. To date, iPD-1 expression has been identified in several solid tumors, with its functional impact differing substantially depending on the tumor context. Specifically, iPD-1 appears to function as a tumor suppressor in NSCLC [144] and colorectal cancer [145]. Conversely, iPD-1 has been shown to promote tumor growth in several malignancies, including melanoma [146], hepatocellular carcinoma [146], pancreatic ductal adenocarcinoma [147], thyroid cancer [148], glioblastoma multiforme (GBM) [149], and triple-negative breast cancer (TNBC) [150]. Notably, iPD-1 is constitutively expressed in tumor cells, distinguishing it from the inducible expression pattern of PD-L1. Nonetheless, its precise biological role remains to be fully elucidated [147]. While emerging evidence highlights the functional relevance of iPD-1, the underlying molecular mechanisms remain poorly understood, warranting further investigation and careful interpretation of its potential therapeutic implications.

In NSCLC, treatment with chemotherapeutic agents such as cisplatin has been shown to increase iPD-1 expression in tumor cells [144]. This phenomenon may be attributed to systemic inflammation triggered by chemotherapy or secondary activation of T cells following treatment with ICIs, leading to increased interferon production, particularly IFN-γ. These cytokines are known to promote the expression of PD-1 ligands (PD-L1/PD-L2) as well as iPD-1 within TME [144]. Proposed molecular mechanisms involve the activation of the mTOR signaling pathway via protein kinase B (PKB/AKT) and extracellular signal-regulated kinases 1 and 2 (ERK1/2), which are components of the MAPK cascade [134,144,145,146]. Additionally, acetylation of the tumor suppressor p53 has been shown to facilitate iPD-1 transcription by enhancing local chromatin acetylation at its promoter region [151]. Increased levels of iPD-1 have been associated with improved therapeutic outcomes following anti-PD-1 treatment, particularly in promoting tumor remission [144]. Nevertheless, the precise role of iPD-1 in modulating treatment response and tumor cell biology remains an active area of research. It may have important implications for the design of future immunotherapeutic strategies [144].

Another mechanism of tumor immune escape involves the secretion of extracellular vesicles (EVs) by cancer cells, which display PD-L1 on their surface. PD-L1 binds to the PD-1 receptor on T cells, suppressing their activation and reducing the number of active T cells within the TME, thereby promoting immunosuppression. However, it remains unclear whether all types of cancer cells are capable of secreting PD-L1-bearing EVs, whether all such EVs contribute equally to immunosuppression, and what the precise mechanisms underlying this form of immune regulation are [72,152].

### 3.3. PD-1 as a Receptor

Two independent ligands, PD-L1 (B7-H1 or CD274) and PD-L2 (B7-DC, CD273), are expressed in distinct cells through different expression patterns, enabling them to fulfill their roles in peripheral organs (PD-L1) and lymphoid organs (PD-L2) [153,154]. Additionally, their expression is regulated by different cytokines: PD-L2 is upregulated by interleukins IL-4 and IL-13 [110], while PD-L1 is regulated by interferon-γ [155].

PD-L1 is expressed over a much broader range than PD-L2 in both hematopoietic and non-hematopoietic cells: in non-lymphoid tissues (such as the heart, placenta, and skeletal muscles) and on APCs, including peripheral blood monocytes, activated dendritic cells (DCs), some cancer cells, and keratinocytes [103]. In contrast, PD-L2 shows two- to six-fold higher affinity for PD-1 [156] and is expressed on activated DCs, macrophages [109], and cultured bone-marrow-derived mast cells [157].

Both ligands are type I transmembrane proteins with characteristic extracellular domains: an IgV-like domain interacting with the PD-1 receptor and an IgC-like domain, transmembrane region, and cytoplasmic domain [112]. PD-L1 consists of 290 amino acid residues, while PD-L2 has 273 residues [19]. Their sequences show 37% homology [110]. The IgV-like domain of the PD-L2 ligand exhibits the classic topology of a β-sandwich formed by the front layer A′GFCC′ and the back sheet ABED, which are connected by an internal disulfide bridge between strands B and F [109]. A similar topology is seen in the IgV-like domain of the PD-L1 ligand, except that the front sheet consists of AGFCC′C″ β-sheet, and the back sheet is BED [158]. When comparing the sequences of PD-L1 and PD-L2, it can be observed that 6 out of the 14 residues involved in binding to PD-1 are conserved for both ligands, and 2 additional residues are analogous (E and D or Q and E in the sequences of PD-L2 and PD-L1) [109]. This similarity suggests an analogous binding mechanism for both ligands [109]. Still, the interactions differ at the molecular details level [159], with structure and biochemical studies of the complexes with PD-1 indicating a 1:1 ligand–receptor binding stoichiometry [19,109].

Upon binding of PD-1 to PD-L1, a small but significant conformational change occurs within the GFCC′ strand and the CC′ loop, forming a hydrophobic surface for the interaction [19]: six residues from PD-1 and five from PD-L1 [160]. One of the key residues responsible for increasing the interaction surface through induced fit is Tyr123 [19]. In the PD-1/PD-L2 interaction, 18 hydrogen bonds are formed by 11 residues from strands C, C′, and G, as well as the CC′ and FG loops from PD-1; 11 residues from strands A, C, F, and G; and the AA′ and FG loops in PD-L2 [109]. Additionally, a small hydrophobic core extending the interaction site is formed from residues in strands C, F, and G; the FG loop in PD-1; and residues in strands F and G and the FG loop in PD-L2 [109]. The approximate center of the PD-1/PD-L2 interaction site is formed by aromatic residues W110 and Y112 in strand G of PD-L2 and the surface of strands C, F, and G of PD-1 [109]. The unique presence of W110 in PD-L2 and its essential role in binding PD-1 suggest that this residue might be responsible for the higher affinity of PD-L2 compared to PD-L1 [19,109].

### 3.4. PD-1’s Inhibitors

Both interaction surfaces between PD-1 and its ligands PD-L1 and PD-L2 are flat, hydrophobic, and devoid of binding pockets, making this surface a challenging molecular target for small-molecule inhibitors [161].

#### 3.4.1. Small Molecules

**Figure 4:**
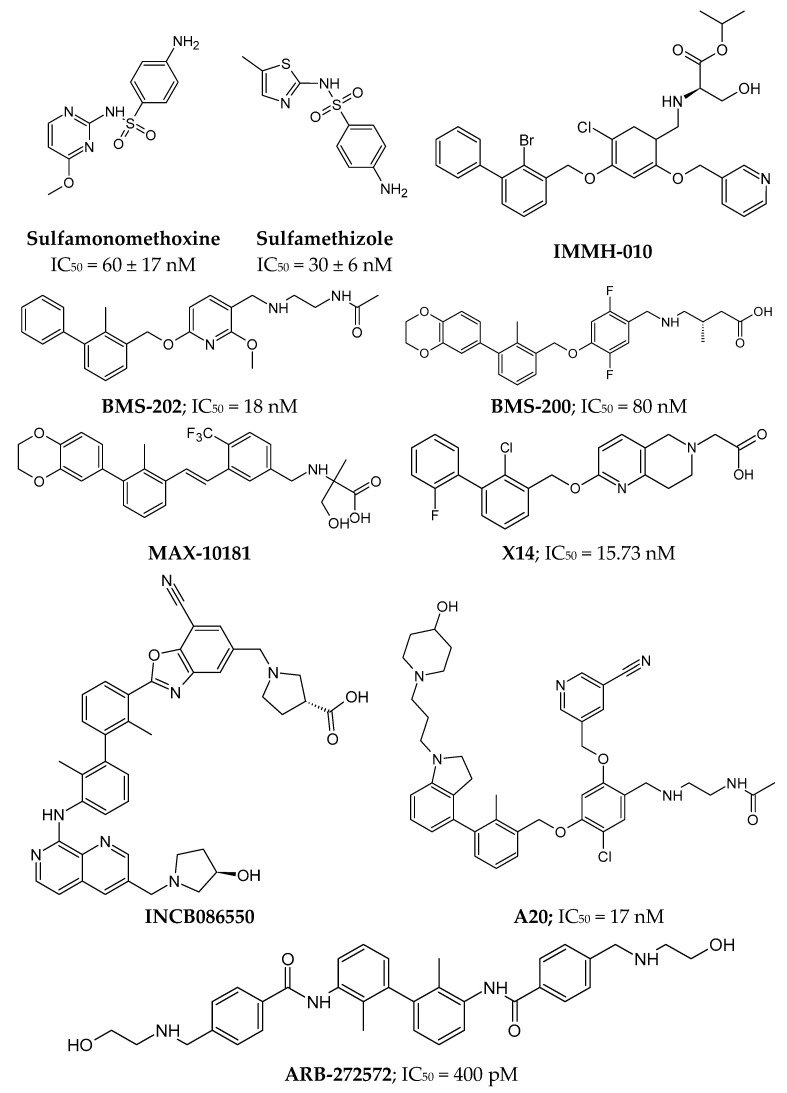
Structures of PD-1 small-molecule inhibitors.

The first sulfonamide derivatives (e.g., **Sulfamonomethoxine** and **Sulfamethizole**, Figure 4) were patented by Aurigene in 2013. They were primarily aimed at compounds interacting with PD-1 for immunotherapy or the treatment and prevention of autoimmune diseases, transplant rejection, infections, or cancers [162]. In the IFNγ-release assay, antagonistic activity was demonstrated in T cells expressing PD-1 [161].

An alternate group of small molecules based on a biphenyl scaffold (e.g., **BMS-202**; see Figure 4) and 2,3-dihydro-1,4-benzodioxinyl (e.g., **BMS-200**; see Figure 4) was a series patented by Bristol Myers Squibb [163]. These inhibitors interact directly with the hydrophobic surface of PD-L1 (including residues Tyr56 and Tyr123, which are critical to the PD-1/PD-L1 interaction), inducing the formation of dimers of these molecules, which can no longer interact with PD-1 molecules [164,165]. BMS-200 induces the formation of PD-L1 homodimers and their conformational changes (the movement of the aromatic ring of Tyr56), creating a hydrophobic tunnel between the molecules [165]. The published compounds show an IC_50_ value at the nanomolar level (homogeneous time-resolved fluorescence, HTRF assays) [165,166].

Based on the scaffold of compounds patented by Bristol Myers Squibb, a series of biphenyl derivatives has been proposed, several of which are currently in clinical trials [167]. In Phase I, there are compounds such as **MAX-10181** (NCT04122339, Figure 4) by Maxinovel Pharma for the treatment of advanced solid tumors [167,168,169] and **IMMH-010** (NCT04343859, Figure 4), developed by Tianjin Chasesun Pharmaceutical Co., LTD., for the treatment of malignant neoplasms [170]. IMMH-010 is a prodrug whose isopropyl ester undergoes hydrolysis to a carboxyl group, producing the active metabolite YPD-29B [170]. Meanwhile, the compound **INCB086550** (NCT04629339, Figure 4), developed by Incyte Corporation, is in Phase II clinical trials for the treatment of NSCLC, urothelial cancer, renal cell carcinoma, hepatocellular carcinoma, and melanoma [171].

Another interesting example of biphenyl derivatives includes compounds with symmetry axis. For instance, the compound **ARB-272572** (Figure 4), developed by Arbutus Biopharma, demonstrated in vivo inhibition of PD-1/PD-L1 signaling by inducing PD-L1 receptor homodimerization (IC_50_ = 400 pM) [172]. This highlights its potential translational relevance beyond in vitro potency. Another derivative is **compound A20** (Figure 4), in which the 2,3-dihydro-1,4-benzodioxinyl group was replaced with a 4-phenylindoline group, resulting in an inhibitor with an IC_50_ of 17 nM [173]. Introducing minor modifications, such as cyclization, also increases inhibitory potential of compound. The **compound X14** (Figure 4), which has an additional naphthyridine ring in its structure, inhibits the PD-1/PD-L1 interaction at an IC_50_ of 15.73 nM, which is more potent than the parent compound BMS-202, which contains a pyridine ring [174].

Another example of a small molecule with potential to inhibit the PD-1/PD-L interaction is the compound CA-170, whose structure was based on the NP-12 peptide [175]. However, additional studies showed that this compound does not directly bind to PD-L1 but instead binds to the VISTA receptor [176], and as such, it is described later in this work.

#### 3.4.2. Peptides

**Figure 5:**
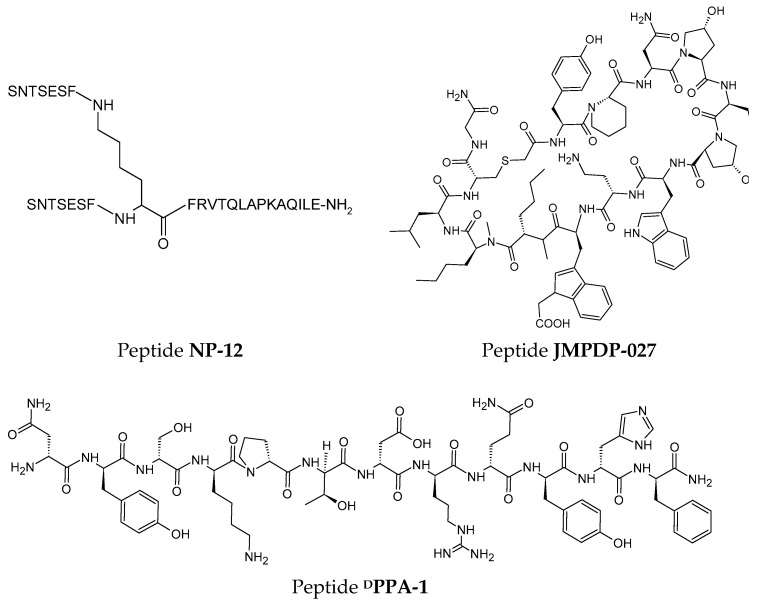
Structures of PD-1 peptide inhibitors.

The rational design concept enabled the development of the first peptide inhibitor of PD-1, the branched peptide **NP-12** (Figure 5) [177]. Aurigene patented the sequence of this peptide, which consists of 29 amino acid residues [177], and is arranged in space to form structural elements of the PD-1 protein [149] to reduce immunogenicity [178].

Initially, it was suggested that peptide NP-12 exhibited similar effects on the PD-L1 and PD-L2 ligands, influencing the proliferation of T cells and exhausted T cells [179]. However, further studies showed that the peptide does not directly bind to PD-L1 but influences the PD-1/PD-L pathway or another T cell activating pathway [176]. Nonetheless, peptide NP-12 affected increasing survival in an immunosuppressive model of sepsis [180] and various syngeneic tumor models [179]. Similarly, based on the crystal structure of the PD-1/PD-L1 complex, a sequence of an unbranched peptide **PL120131** (Ac-GADYKRITVKVN-NH_2_) was proposed, which containing residues responsible for interaction with the PD-L1 ligand, namely, amino acids from Gly120 to Asp131 [181]. The peptide PL120131 showed affinity to PD-1 with a K_D_ of 305 ± 1.55 nM, and in biological studies on Jurkat cells, the peptide was shown to enter the TME, interact, and induce cancer cell death [181].

The next group of peptides acting as inhibitors of the PD-1/PD-L1 interaction consists of cyclic peptides patented by Bristol Myers Squibb in 2014 [177,182]. The best-known sequences are **BMS-57** (cyc(Phe-NMeAla-Asn-Pro-His-Leu-Ser-Trp-Ser-Trp-NMeNle-NMeNle-Arg-Cys)-Gly) and **BMS-71** (cyc(Phe-NMePhe-NMeNle-Sar-Asp-Val-NMePhe-Tyr-Sar-Trp-Tyr-Leu-Cys)-Gly), which consist of 15 and 14 amino acid residues, respectively [183]. Unlike the peptide NP-12, the macrocyclic systems did not rely on the sequence and spatial conformation of PD-1 but instead on the creation of a hydrophobic interaction surface through the presence of a large number of hydrophobic canonical amino acids, N-methylated derivatives, and unnatural amino acid residues [179]. For the most active peptides, BMS-57 and BMS-71, crystal structures were obtained in complex with PD-L1, confirming that the binding area of the macrocyclic molecules to PD-L1 is the same as that of PD-1. Still, the residues responsible for interaction differ [184]. In contrast to the small-molecule inhibitors of the BMS series, binding of the macrocyclic peptides does not induce conformational changes or dimerization of PD-L1 [184].

Another macrocyclic peptide that inhibits the PD-1/PD-L1 interaction is peptide **pAC65** (cys(Ac-Trp-NMeAla-Asn-Pro-Dap-Leu-Hyp-Trp-Dab-TrpNAc-NMeNle-NMeNle-Cys)-Gly-NH_2_), also patented by Bristol Myers Squibb in 2018 [183]. It deserves special mention due to its activity in the subnanomolar range (IC_50_ = 1.80 ± 0.16 nM by HTRF) and the potential for PD-1 expression on T lymphocytes, similar to that of antibodies used in clinical practice (atezolizumab, avelumab, and durvalumab) [185]. The peptide pAC65 binds to PD-L1 in a 1:1 molar ratio at the same site as PD-1, engaging β-sheets GFCC’ and, among other residues, Tyr123 within the hydrophobic interaction surface [185].

The rational design methodology was also helpful in developing a derivative of BMS-57. Conformational analysis identified steric hindrances between residues Phe1, NMeNle12, Cys14, Gly15, and the hydrophilic part of Arg13 [186]. By conducting several optimization series in MD simulation, modifications were introduced to create a derivative without conformational limitations [186]. For example, replacing Arg13 with leucine restored of the Phe1 conformation to its physiological range, and replacing Phe1 with Tyr allowed the formation of an additional hydrogen bond with Ser117 of PD-L1 [186]. The best parameters were exhibited by peptide **JMPDP-027** (Figure 5), which demonstrated high resistance to proteolysis due to the introduction of non-canonical amino acid residues, lacked toxicity, and showed strong anticancer activity in vivo in a colon carcinoma model [186].

The following approach to designing inhibitors uses of known tertiary structures of mini proteins, incorporating residues that interact with the molecular target into their sequence. In this way, the peptide inhibitor **MOPD-1** was designed using a described scaffold comprising 46 amino acids, three internal disulfide bridges, and a tertiary structure consisting of an α-helix and three β-strands (PDB: 5JG9-gEHEE [187]) [188]. The conformation of this mini protein mimics the surface of the PD-1 protein, similar to the macrocyclic peptide of the NP-12 type [188], with the difference that the introduced residues interacting with PD-L1 were based on a PD-1 structure optimized for affinity [189]. This inhibitor is characterized by stability in serum and the ability to inhibit the growth of CT26 cancer tumors in mouse models, with a K_D_ value of 0.3 μM [188].

A succeeding series of designed inhibitors of the PD-1/PD-L1 interaction are peptides based on the engrailed homeodomain (ENH) mini protein structure, a stable system of three α-helices [187]. Among the 32 sequences, the last one, numbered 32 (**peptide 32**, AFSSEQEKWLKLWFKKLRYLTEWHRKKLSSELGLNEAQIKIWFQNK), demonstrates a relatively high melting temperature and the highest affinity for PD-L1, with a K_D_ = 51.4 nM [190]. It is worth noting that, compared to previously described peptides, this is the first tertiary structure stabilized solely by hydrophobic interactions rather than covalent bonds—disulfide bridges [190].

Another method for discovering new peptide inhibitors is phage display technology, which identifies peptides that interact with a specific molecular target. The peptide **CLP002** (WHRSYYTWNLNT) was proposed using this approach, exhibiting a K_D_ value of 366 ± 150 nM against PD-L1. The binding site of this inhibitor on PD-L1 significantly overlaps with the binding residues in the PD-1/PD-L1 complex [191]. Similarly, using bacterial phage display methodology, the peptides **TPP-1** (SGQYASYHCWCWRDPGRSGGSK) [192] and **PBP** (SGQQRTSADCWEDHGWGSGGSK) [193] were identified, both constructed entirely from natural amino acid residues. The affinity of the **TPP-1** peptide for PD-L1 was significantly better, with a K_D_ = 95 nM [192], while for the **PBP** peptide, the K_D_ value was 17.44 μM [193].

A challenge in using peptides as drugs is their metabolic instability, which can be improved by incorporating unnatural amino acids into the sequence. However, a downside of such molecules may be the potential production of antibodies against the peptide [179]. Despite this, the literature includes reports on using the peptide **^D^PPA-1** (Figure 5), a PD-L1 antagonist, loaded in nanoparticles for photodynamic and photothermal therapy in breast cancer and lung metastasis [194,195].

The final approach to designing peptide inhibitor sequences is de novo design, which, like the previous methodologies, focuses on identifying and arranging the amino acid residues responsible for binding to the PD-L1 ligand and then combining them into a three-dimensional structure based on known constructs [196]. The best of the proposed peptides, **Ar5Y_4** (GNWDYNSQRAQLYNQ), showed micromolar affinity for PD-1, with a K_D_ = 1.38 ± 0.39 μM [196].

#### 3.4.3. PROTACs and LYTACs

**Figure 6:**
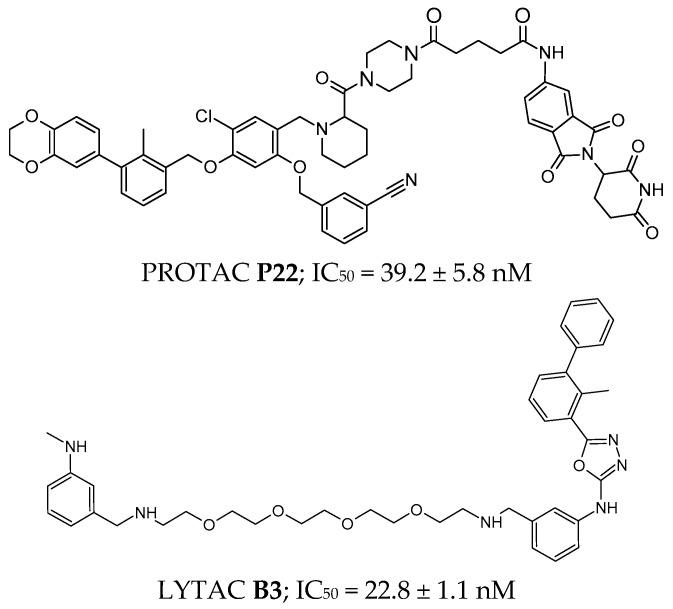
Structure of PD-1 PROTAC and LYTAC.

An example of a PROTAC molecule is **P22** (Figure 6), which inhibits the PD-1/PD-L1 interaction at an IC_50_ level of 39.2 ± 5.8 nM [197]. The fragment interacting with the E3 ubiquitin ligase is a pomalidomide derivative, the linker is a piperazine derivative, and the part interacting with PD-L1 is the BMS-1198 molecule with a 2,3-dihydro-1,4-benzodioxinyl ring [197].

In a similar way to PROTACs, lysosome-targeting chimeras (LYTACs) can be obtained, with the difference that a lysosome-targeting receptor (LTR) is engaged for degradation instead of an E3 ubiquitin ligase [198]. An example of this type of molecule is **compound B3** (Figure 6), which exemplifies this class, demonstrating potent inhibition of the PD-1/PD-L1 interaction with an IC_50_ = 22.8 ± 1.1 nM [199]. Importantly, in vitro studies confirmed PD-L1 degradation activity, and in vivo experiments showed significant tumor growth inhibition at a dose of 120 mg/kg, highlighting its translational potential [199].

The literature also describes PROTAC peptides, which, in addition to the standard fragments, contain a cell-penetrating peptide of eight arginine residues [200]. The molecular targets of **PROTAC-peptide 1** (YRCMISYGGADYKCIT-GSGS-ALAPYIP-RRRRRRRR) and **PROTAC-peptide 2** (CGIQDTNSKKQSDTHLEET-GSGS-ALAPYIP-RRRRRRR) were PD-1 and PD-L1, respectively, and the expression levels of these proteins significantly decreased at doses <5 μM [200]. 

## 4. VISTA

### 4.1. VISTA Structure

VISTA is another example of a negatively regulated checkpoint in the immune system. The V-domain Ig suppressor of T cell activation (VISTA) [201], also known as C10orf54 [202], PD-H1 [203], DD1α [204], Gi24 [205], Dies1 [206], or SISP1 [207], is a type I transmembrane protein (55–65 kDa, 279 amino acid residues), which can be divided into three components: the longest fragment being a single N-terminal IgV-like domain that is glycosylated [208], a transmembrane domain, and a 95-amino acid cytoplasmic tail that interacts with factors initiating signaling cascade pathways [201,203]. Four different research groups have published the crystal structure of human VISTA [209,210,211,212].

The extracellular domain adopts a canonical β-sandwich structure with ten antiparallel β-strands, differing from the standard topological structure of the B7 family fold by the presence of an additional strand H [209,212]. It shows approximately 23% homology to PD-L1 [5,212]. The domain contains six cysteine residues that form three disulfide bridges between the β-strands—B-F (Cys22-Cys114), A’-H (Cys12-Cys146), and CC’-F (Cys51-Cys113) [209,212]. All these bonds stabilize the VISTA structure, with the bridge formed between strands A’-H enforcing a shortening of the stem region to about nine amino acids and limiting the rotation of the entire protein relative to the cell membrane, distinguishing VISTA from other IgV-like domains [209,212].

Another unique feature of the sequence in VISTA is the high accumulation of histidine residues in the extracellular domain [209]. These residues account for 8.6% of the extracellular residues compared to other type I extracellular domains, which have only 2.4% [210]. Compared to orthologs, most histidines in VISTA are conserved, which may account for its unique functions [210]. The residues H153, H154, and H155 located between strands G and F are essential for interactions with VISTA binding partners at different pH levels [210,212].

Additionally, the cytoplasmic fragment of VISTA is similar to that of members of the CD28 family (PD-1 and CTLA-4) [211,213]. At the C-terminus, several signaling motifs can be distinguished: motifs binding to Src homology domain 3 (SH3) and Src homology domain 2 (SH2), as well as a specific sequence for protein kinase C (PKC) and for casein kinase 2 (CK2) [213,214]. Because of the presence of these binding regions, VISTA can simultaneously function as both a receptor and a ligand [201,203,213,215,216].

### 4.2. VISTA Expression in Health and Disease

VISTA is expressed in both hematopoietic and non-hematopoietic cells. Typically, the highest expression occurs in brain [217], stomach, and thyroid cells [218]. Moderate expression is found in the spleen and liver [213,219], while it is relatively low in the bones and heart [207,220]. VISTA is primarily strongly expressed in mature APCs [221] derived from myeloid lineage [202,222] and to a lesser extent in T cells and Tregs [219,223]. In activated CD4+ T cells, the level of VISTA expression is higher than in activated CD8+ T cells [219,223,224,225]. VISTA can also be expressed as soluble VISTA-Ig fractions that inhibit T cell proliferation [226,227]. The transcription factors that regulate VISTA expression are not well understood. In inflammatory conditions, VISTA is not transcribed due to the blocking of its promoter by factors such as NF-κB, Fos, and JunD [5,228,229]. In the case of cancer cells, VISTA transcription occurs due to an increased amount of p53 [204,230] and hypoxia-inducible factor 1-alpha (HIF-1α) [231] proteins binding to the VISTA promoter [223]. Additionally, BRAF binds to FOXP3+, neutralizing its inhibitory effect [224,231,232].

VISTA plays a crucial role in regulating immune system homeostasis, particularly in the context of immune responses [233,234]. Its immunomodulatory functions are complex and involve both APCs and T cells, making the mechanisms of its action challenging to define clearly [5]. In the context of naive CD4+ T cells, VISTA acts as an inhibitor of their activation in situations where no foreign antigens are present [234,235]. This action aims to prevent unwanted autoimmune reactions, which is supported by observations of reduced levels of VISTA in autoimmune diseases such as rheumatoid arthritis, systemic lupus erythematosus, and multiple sclerosis [5,236,237,238]. VISTA also influences the regulation of chemokine expression and their receptors in myeloid cells, such as macrophages and monocytes, which may have significant implications for inflammatory and immune responses [239,240]. Additionally, VISTA suppresses the antigen presentation process in APCs, which can affect T cell activation [5].

Initially, VISTA was identified mainly in tumor-infiltrating lymphocytes. Later studies revealed its presence in several types of cancer, including bladder [241], gastric [242,243], ovarian [225,244,245,246,247], melanoma [248,249], pancreatic [250,251,252], breast [253,254,255], acute myeloid leukemia [256], and colorectal carcinoma [202]. Excessive expression of VISTA can have different consequences depending on the type of cancer, suggesting that its role in cancer pathogenesis is complex and requires further investigation [5,257]. Combining therapies that inhibit VISTA with other ICs show promising results in enhancing antitumor responses in various types of cancer [258]. This approach may also help prevent the development of therapy resistance, which is crucial in cancer treatment [227,255,259,260,261].

However, many questions remain regarding the molecular mechanisms and signaling pathways that VISTA utilizes in different cell types [258]. Further research is necessary to better understand these processes and their potential implications in cancer therapy and autoimmune diseases [262].

### 4.3. VISTA as a Receptor and a Ligand

The fundamental difference in the action of VISTA as a ligand and as a receptor results from the type of cells in which it is expressed [5,263]. In T cells, VISTA functions as a receptor that interacts with ligands, inhibiting these cells’ activity and further transmitting and amplifying signals [227,264]. In contrast, VISTA acts as a ligand in non-T cells, such as APCs or cancer cells, binding to other T cell receptors [5]. The best-known receptors for VISTA are VSIG-3 and PSGL-1 [254]. Less well-characterized receptors that warrant further investigation include Gal-9, VSIG8, MMP-13, Sdc-2, LRIG1 [265], and IGSF11 [266].

VSIG-3 is a single-pass (type I) transmembrane protein belonging to the immunoglobulin superfamily. It consists, similar to the VISTA receptor, of an N-terminal domain (IgV-like and Ig-like C2-type domains), a transmembrane region, and a C-terminal PDZ domain [267]. VSIG-3 is expressed in specific non-hematopoietic cells, such as the brain, testes, ovaries, adrenal glands, kidneys, skeletal muscles, and thyroid [267,268]. Since it is not present in all body cells, it represents a potential target for immunotherapy with fewer side effects [5]. It is an adhesion molecule that regulates cell aggregation [268,269].

In the context of cancer cells, its presence has been observed in breast, colorectal, gastric, and hepatocellular carcinoma, suggesting that VSIG-3 may promote the growth and proliferation of cancer cells [267]. The interaction between VSIG-3 from tumor cells and VISTA from T cells activates an unfavorable regulatory pathway, leading to a reduction in T cell proliferation and immune cell infiltration, as well as an increase in the production of proinflammatory cytokines and chemokines, including IL-2, IL-17, and IFN-γ [223,245,268,270,271,272]. VSIG-3 binds to VISTA at a neutral pH (7.4) with conserved C-C′ loop interaction [272].

P-selectin glycoprotein ligand-1 (PSGL-1) is a homodimeric type I transmembrane glycoprotein connected by disulfide bridges, with each monomer consisting of three domains: an extracellular domain, a transmembrane domain, and a cytoplasmic domain [269,273,274]. It is widely expressed in hematopoietic cells, such as peripheral T cells, monocytes, neutrophils, and platelets [210], as well as in activated endothelial cells, epithelial cells of the fallopian tubes, and microvascular endothelial cells [211,269].

PSGL-1 interacts with L- and E-selectins as an adhesion molecule that mediates the transport of leukocytes, binding to ligands in infected tissues [275,276]. It stimulates the production of cytokines in T cells, DCs, and macrophages in response to pathogens, including viruses and bacteria [273,275]. PSGL-1 is also classified as a checkpoint molecule in the immune system, inhibiting antitumor responses and facilitating the migration of cancer cells [275].

PSGL-1 interacts with VISTA in the acidic pH (5.85–6.5) found in the TME [210]. At this pH, the histidine residues of VISTA are protonated and susceptible to binding with negatively charged glutamic acid residues and sulfate groups attached to tyrosine residues [210,211]. This interaction may play a significant role in modulating immune responses within the TME, potentially influencing tumor progression and immune evasion [268].

### 4.4. VISTA’s Inhibitors

Even though the ligands and mechanisms of action of VISTA still require further understanding, the literature already includes described small molecules and peptides that interact with VISTA and are involved in stimulating the immune system. 

#### 4.4.1. Small Molecules

**Figure 7:**
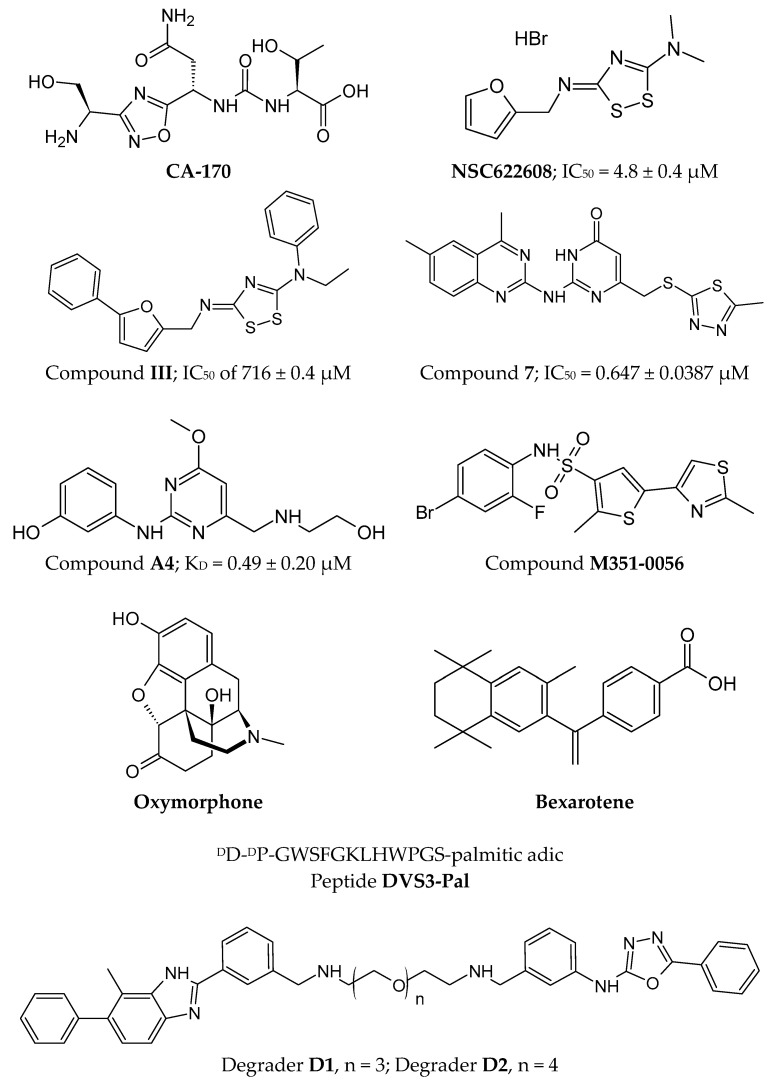
Structures and sequence of VISTA small-molecule, peptide, and PROTAC inhibitors.

One of the first inhibitors of the VISTA/VSIG-3 interaction was the compound **CA-170** (Figure 7), which is currently in Phase II clinical trials for lung, head, and neck/oral cancers, as well as Hodgkin lymphoma and MSI-H positive cancers [277]. Initially described as a PD-1/PD-L1 inhibitor, NMR and HTRF analyses did not confirm such an interaction [176]. CA-170 is an orally bioavailable small molecule that is relatively polar, which affects its exposure to polar solvents and molecular targets [277,278].

Based on the known structure of the CA-170 molecule, attempts were made to design other small molecules that interact with VISTA and its ligands. Using the National Cancer Institute Developmental Therapeutics Program compound database, a FRET-based HTS was conducted, which identified the compound **NSC622608** (Figure 7) with potential interaction with VISTA (IC_50_ = 4.8 ± 0.4 μM) [279]. To develop derivatives of the compound NSC622608 with greater affinity for VISTA, potential inhibitor binding sites were proposed based on the crystal structures of VISTA [279]. Among the 32 proposed structures, **compound III** (Figure 7) demonstrated the highest affinity for VISTA with an IC_50_ of 716 ± 0.4 μM [279].

Based on the CA-170 structure, small-molecule probes labeled with the radioactive isotope ^68^Ga have also been proposed [280]. These probes can be used to visualize and differentiate VISTA expression in various cancer cells [280].

Another example of a first-in-class inhibitor of the VISTA/VSIG-3 interaction is **compound 7** [281,282] (Figure 7), as well as a VISTA degrader, which is a bifunctional molecule that demonstrates significant efficacy in inducing the immune system in the presence of cancer cells [281]. When comparing compound 7 (binding constant of 0.647 ± 0.0387 μM for VISTA) with compound III, similarities between the two molecules are evident, particularly the presence of a heterocyclic ring and aromatic rings. However, compound 7 is a more polar molecule due to additional nitrogen atoms [279,281,282]. Studies conducted on the mechanism of VISTA degradation have shown that this process is autophagy-dependent and indicated by an increase in the expression of LC3II, which is a marker of the autophagosome membrane [281].

Based on the structures of compound **7** and compound III, optimization of a methoxy-pyrimidine-based VISTA small-molecule inhibitor was optimized using molecular docking [283]. The proposed **compound A4** (Figure 7) exhibited a strong affinity with the human VISTA protein (K_D_ = 0.49 ± 0.20 μM) [283].

Theoretical considerations were also conducted based on virtual screening of FDA-approved anticancer drugs (a database of 2315 molecules) as potential interactors with VISTA and another protein, HDAC6 (histone deacetylase), which regulates, among other tubulins [284]. These considerations led to the identification of two drugs from the DrugBank database—**Bexarotene** and **Oxymorphone** (Figure 7)—that theoretically exhibit the strongest binding to VISTA [284]. Similarly, three additional molecules were identified from the PubChem database (PubChem ID: 14187087, 3861164, 6494266), which show potential for forming hydrogen bonds with the arginine residues of VISTA (Arg54 and Arg127) [285]. To confirm these results, additional in vitro and in vivo studies are recommended [284,285].

In addition to VISTA/PSGL-1 or VISTA/VSIG-3 interaction inhibitors, the literature has also described a small-molecule compound modulating VISTA in inflammatory contexts, which may be promising for the treatment of autoimmune diseases [286]. A compound named **M351-0056** (Figure 7) was identified in the ChemDiv library screened using molecular docking and virtual screening as having a high affinity for VISTA and modulating its function likely through the JAK2-STAT2 pathway [286].

#### 4.4.2. Peptides

Phage display screening of VISTA-binding peptides, molecular dynamics simulations using Molecular Operating Environment (MOE), and alanine scanning have been employed to optimize the peptide sequence as VISTA/PSGL-1 inhibitor [287]. From the perspective of the VISTA/PSGL-1 complex, this interaction occurs at a slightly acidic pH (pH 6.0), as PSGL-1 can only bind to VISTA and transmit signals under acidic conditions. The introduction of D-amino acids at the N-terminus of the DVS3-Pal sequence (Figure 7) and the addition of positively charged palmitic acid at the C-terminus significantly increased the peptide’s proteolytic resistance [287].

#### 4.4.3. PROTACs and LYTACs

To know the structure of the VISTA/VSIG-3 interaction inhibitor, a bifunctional small-molecule structure was proposed as a PROTAC [28,199]. One part of the molecule interacts with VISTA, while the other, connected by a linker, mediates lysosome-dependent degradation [199]. The **degraders D1 and D2** (Figure 7) demonstrated in vitro activity in removing VISTA protein from HepG2 cells [199].

## 5. BTLA

### 5.1. BTLA Structure

B and T Lymphocyte Attenuator (BTLA) is a transmembrane glycoprotein of approximately 32 kDa, belonging to the CD28 superfamily [288] and functioning as an immune response inhibitor. Structurally, it consists of three main domains: extracellular, transmembrane, and cytoplasmic [289,290].

The extracellular domain contains a single immunoglobulin-like IgC domain, which enables binding to its ligand, Herpesvirus Entry Mediator (HVEM), which is a member of the tumor necrosis factor (TNF) receptor superfamily. This interaction transmits an inhibitory signal that affects the activity of T and B lymphocytes and other immune cells. The transmembrane domain of BTLA anchors the protein in the cell membrane, ensuring its stabilization and proper functioning. The cytoplasmic domain contains two key motifs: ITIM and ITSM [288]. Upon activation of BTLA by HVEM, these motifs undergo phosphorylation, leading to the recruitment of the tyrosine phosphatases SHP-1 and SHP-2. These enzymes remove phosphate groups from signaling molecules, inhibiting lymphocyte activation and suppressing the immune response [291,292].

BTLA is homologous to other inhibitory receptors, such as PD-1 and CTLA-4, but its ability to interact with HVEM and its signaling mechanism differs. Its structure makes BTLA a crucial immune system regulator, and its function can be modulated in the context of cancer immunotherapy and autoimmune disease treatments [292,293,294].

### 5.2. BTLA Expression in Health and Disease

BTLA is key in inhibiting the immune response, preventing excessive immune system activation. Its functions include inhibiting the activation and proliferation of T and B lymphocytes, reducing the production of proinflammatory cytokines such as IFN-γ and IL-10, as well as regulating immune tolerance and preventing autoimmune diseases [295]. BTLA is an inhibitory receptor whose expression plays a key role in regulating the immune response in both healthy and diseased individuals. BTLA is present on the surface of various immune cells, including T and B lymphocytes, NK cells, macrophages, and DCs. Its expression is particularly high in lymphoid organs such as the spleen, lymph nodes, and thymus. Under physiological conditions, BTLA functions as an immunosuppressive regulator, helping to maintain a balance between activation and immune tolerance. Its interaction with HVEM inhibits excessive lymphocyte activation, preventing the development of autoimmune diseases and chronic inflammatory conditions [6,296].

BTLA is overexpressed in various cancers, leading to an impaired antitumor response. In B-cell lymphomas, such as chronic lymphocytic leukemia (CLL) and diffuse large B-cell lymphoma (DLBCL), as well as in T cell tumors, BTLA is frequently expressed on tumor-derived lymphocytes and is associated with a poor prognosis. A similar phenomenon is observed in lung cancer and melanoma, where BTLA is overexpressed on tumor-infiltrating T cells, impairing their cytotoxic function. In hepatocellular carcinoma, blockade of the BTLA-HVEM pathway increases lymphocyte IFN-γ production, suggesting that BTLA inhibits the antitumor response [297,298,299].

BTLA expression is, therefore, crucial for maintaining immune system homeostasis. Its elevated levels in cancer promote immune evasion by tumor cells, while its reduced expression in autoimmune diseases increases the risk of uncontrolled lymphocyte activation. Understanding the mechanisms regulating BTLA expression may be essential for developing new therapeutic strategies in oncology and treatment of autoimmune diseases [295,300].

### 5.3. BTLA as a Receptor

BTLA is an inhibitory receptor belonging to the CD28 superfamily, playing a key role in regulating the immune response. Its structure comprises an extracellular domain containing a single IgC domain, a transmembrane domain, and a cytoplasmic domain that contains ITIM and ITSM motifs. These structural components enable BTLA to function as a negative regulator of lymphocyte activation, preventing excessive immune responses [301,302].

The primary ligand for BTLA is HVEM, which is a member of the TNF receptor superfamily. The BTLA-HVEM interaction can occur in a cis configuration when both molecules are present on the same cell or in a trans configuration when they are located on different cells. Upon ligand binding, phosphorylation of the ITIM and ITSM motifs in the cytoplasmic domain of BTLA occurs, leading to the recruitment of the tyrosine phosphatases SHP-1 and SHP-2. These enzymes remove phosphate groups from signaling proteins involved in lymphocyte activation, inhibiting their proliferation, reducing the production of proinflammatory cytokines, and weakening effector functions [303]. BTLA preferentially binds to the stronger phosphatase SHP1, inhibiting CD28 and TCR signaling pathways essential for T cell activation [72]. The interaction between BTLA and its ligand HVEM suppresses T cell activation and activates the NF-κB signaling pathway, promoting the maturation of APCs. This unique bidirectional signaling between an IC and its ligand underscores the context-dependent and dual regulatory roles of BTLA and HVEM in immune modulation [6].

Like other inhibitory receptors, such as PD-1 and CTLA-4, BTLA plays a crucial role in maintaining immune homeostasis. Its activity prevents excessive activation of T and B lymphocytes, protecting the body from autoimmunity and chronic inflammation. In the context of cancer, BTLA promotes immunosuppression, which can weaken the antitumor immune response. Conversely, reduced BTLA expression in autoimmune diseases contributes to excessive lymphocyte activation and tissue damage within the body [304,305].

### 5.4. BTLA’s Inhibitors

Because of its role in inhibiting the immune response, BTLA is a potential therapeutic target. Blocking its function may enhance the antitumor response, similar to that of PD-1 or CTLA-4 inhibitors, while activating it may be applied in treatment of autoimmune diseases, transplantation, and sepsis. BTLA is thus a key regulator of the immune response, whose expression and function can be modulated to treat both cancer and autoimmune diseases [306].

#### Peptides

The research on peptide inhibitors of the BTLA–HVEM complex has focused on the design and synthesis of short peptides capable of blocking BTLA–HVEM interactions. This has taken advantage of protein sequences such as herpesvirus glycoprotein D (gD), which naturally binds to HVEM and cysteine-rich domains (CRDs) of HVEM proteins that interact with BTLA [307].

**Figure 8:**
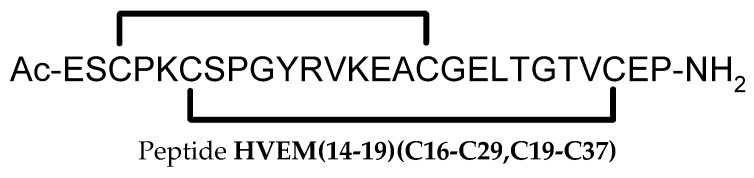
Sequence of BTLA peptide inhibitor.

In particular, the peptide **HVEM(14-19)(C16-C29,C19-C37)** (Figure 8) was shown to bind to BTLA 2.45 times more strongly than the native HVEM protein, making it one of the most potent inhibitors of the BTLA–HVEM complex. The peptide is based on the CRD1 sequence of the HVEM protein, which is responsible for its interaction with BTLA. The introduction of disulfide bonds into its structure stabilizes the β-hairpin conformation, thereby increasing its affinity for the target. In bioassays, the HVEM(14-19)(C16-C29,C19-C37) peptide effectively blocks the formation of the BTLA–HVEM complex and could potentially restore T cell immune function. In addition, several peptides based on the gD sequence, such as gD(1-36)(K10C-T29C) and gD(1-38)(L4C-V37C), effectively blocked BTLA–HVEM interaction while not interfering with HVEM’s interaction with LIGHT, which is crucial for maintaining its immune system-stimulating function [307,308,309]. In vitro experiments conducted on isolated T lymphocytes showed that some of the peptide inhibitors of the BTLA–HVEM complex exhibit a pronounced immunomodulatory effect. The increased expression of activation markers (CD69, CD25), an increase in lymphocyte proliferation, and changes in immune memory cell subpopulations were observed, suggesting its potential use as an immunomodulator in anticancer therapy and the treatment of autoimmune diseases [308,310].

## 6. TIM-3

### 6.1. TIM-3 Structure

T cell immunoglobulin and mucin-domain containing protein 3 (TIM-3) is a type I surface glycoprotein whose structure includes a mucin domain, an amino-terminal IgV-like domain with five non-canonical cysteines, a transmembrane domain, and a cytoplasmic tail. Structurally, it belongs to the TIM family, which includes three proteins in humans: TIM-1, TIM-3, and TIM-4 [311].

TIM-3 is a protein with a distinct structure that plays a crucial role in immune regulation and tolerance. It contains an IgV-like domain, which consists of an antiparallel β-sheet stabilized by disulfide bridges formed by non-canonical cysteines. This domain is further stabilized by two hydrogen bonds, a common feature in immunoglobulin superfamily (IgSF) domains, formed by Trp-53 and Tyr-109. Additionally, a salt bridge contributes to the stability of the domain. A significant structural feature of TIM-3 is the “cleft” formed by the CC′ and FG loops, which are stabilized by hydrogen bonds and ionic interactions. This unique surface structure is essential for ligand binding, especially with phosphatidylserine. TIM-3 has the smallest mucin domain among the TIM family proteins. This region is rich in proline, serine, and threonine, contributing to its structural properties. The transmembrane region consists predominantly of hydrophobic amino acids, allowing the protein to anchor within the lipid bilayer of the cell membrane and extend into the intracellular space [312].

### 6.2. TIM-3 Expression in Health and Disease

TIM-3 is an immunoreceptor found on various immune cells, including Th1 and Th17 lymphocytes, cytotoxic T cells (CD8+), monocytes, macrophages, NK cells, and DCs. Its expression is dynamically regulated in response to the physiological and immune environment. This receptor plays a crucial role in regulating immune responses, including immune tolerance, T cell apoptosis, and CD8+ T cell differentiation through the regulation of the mTORC1 pathway. TIM-3 expression is induced by the transcription factor T-bet, which is activated by cytokines such as IL-12 and IL-27 [313,314,315,316].

TIM-3 binds several ligands, including galectin-9, CEACAM1, phosphatidylserine, and HMGB1. Interaction with galectin-9 leads to apoptosis of Th1 lymphocytes and suppression of Th1 and Th17 responses, while binding to CEACAM1 inhibits CD8+ T cell activity [317,318]. Phosphatidylserine allows for the recognition of apoptotic cells, and interaction with HMGB1 weakens innate immune activation by DCs [319].

TIM-3 expression is significantly increased and often co-expressed with other IC receptors such as PD-1 or LAG-3 in the context of cancer and chronic infections. This leads to the functional exhaustion of T and NK cells, reducing their ability to eliminate cancer or infected cells. In tumors, including glioblastoma, colorectal cancer, lung cancer, and thyroid cancer, high TIM-3 expression is associated with disease progression, metastasis, and a poor prognosis. Additionally, TIM-3 is present in immunosuppressive cells such as Tregs and M2 macrophages, contributing to disease progression. In autoimmune diseases, TIM-3 expression supports the suppressive function of Tregs, helping to control excessive immune activation. In chronic infections such as HBV, high TIM-3 expression on T cells correlates with their exhaustion and loss of effector function [313,317,320].

TIM-3 exhibits complex biological activity within TME. The inhibition of this receptor occurs through interactions involving the tyrosine kinase FYN and the human leukocyte antigen B-associated transcript 3 (BAT3) with the cytoplasmic tail of TIM-3. This binding modulates the activity of lymphocyte-specific protein tyrosine kinase (LCK), thereby maintaining T cell function. Upon ligand binding, tyrosine residues on TIM-3 become phosphorylated, leading to the release of BAT3. This event enables the recruitment of FYN kinase and the subsequent activation of T cells via the activation of PAG1 (phosphoprotein associated with glycosphingolipid-enriched microdomains 1), which recruits the CSK kinase and facilitates LCK phosphorylation [72,321]. An alternative proposed mechanism involves the interaction of the extracellular domain of TIM-3 with phosphatidylserine (PS), which can activate TCR signaling pathway. However, TIM-3 aggregation induced by galectin-3 prevents the binding of TIM-3 to PS, thereby inhibiting NK cell activity and cytokine production [72,322].

### 6.3. TIM-3 as a Receptor

The TIM-3 is a surface receptor that plays a crucial role in regulating immune responses. It belongs to the TIM family, which comprises three members in humans (TIM-1, TIM-3, and TIM-4) and eight members in mice. TIM-3 is a type I transmembrane protein, with a structure comprising an immunoglobulin (IgV) domain rich in cysteines, a mucin-like domain, a transmembrane domain, and a cytoplasmic tail. Its primary function is to modulate the activity of T lymphocytes and other immune cells, making it a critical IC.

The IgV domain of TIM-3 consists of antiparallel β-sheets stabilized by disulfide bonds, which provide its characteristic structure [323]. A key feature of this domain is a groove formed by the CC′ and FG loops, where ligand binding occurs. These ligands, including galectin-9 (Gal-9), CEACAM1, PS, and HMGB1, trigger specific biological effects upon receptor interaction. Galectin-9 induces apoptosis in Th1 lymphocytes, leading to immunosuppression [324]. CEACAM1 enhances TIM-3 function by forming heterodimers that inhibit CD8+ T cell activity [325]. PS binds to TIM-3, enabling apoptotic cell recognition that may influence NF-κB pathway activation, while HMGB1 regulates innate immune responses by affecting DC function [326].

Unlike classical immune receptors, TIM-3 does not contain ITIM sequences in its cytoplasmic tail, which is typical of inhibitory receptors. Instead, it possesses five conserved tyrosine residues that undergo phosphorylation upon ligand binding, activating signaling pathways that suppress the effector function of T lymphocytes and NK cells. Under physiological conditions, TIM-3 functions as a regulator of immune tolerance, preventing excessive immune activation and limiting inflammation. Its expression is induced under chronic antigenic stimulation, leading to T cell exhaustion and reduced cytokine production, such as IFN-γ and TNF-α [327,328,329].

TIM-3 is crucial in tumor progression and immunosuppression associated with chronic infections. Its overexpression on CD8+ T cells and NK cells within the TME leads to the functional exhaustion of these cells and weakened antitumor responses. Furthermore, TIM-3 is often co-expressed with other ICs, such as PD-1 and LAG-3, further enhancing its immunosuppressive effects. For this reason, TIM-3 is currently being investigated as a therapeutic target in modern immunotherapy strategies, including blocking its interaction with ligands using mAbs or small-molecule inhibitors [311,323].

### 6.4. TIM-3’s Inhibitors

Research focuses on developing various classes of TIM-3 inhibitors, including mAbs, peptide-based inhibitors, and small-molecule compounds. While mAbs have shown efficacy in blocking TIM-3 interactions, peptide and small-molecule inhibitors offer improved tissue penetration, better TME accessibility, and potentially lower production costs. These alternative strategies enhance antitumor immune responses by restoring T cell activity and overcoming resistance to other checkpoint inhibitors, such as PD-1/PD-L1 blockers [330,331,332].

#### 6.4.1. Small Molecules

Small-molecule TIM-3 inhibitors represent a novel and promising approach in cancer immunotherapy, aiming to overcome resistance to ICI, such as PD-1/PD-L1 blockers. TIM-3, as a critical IC, is highly expressed in various immune cell populations, including CD4+ and CD8+ T lymphocytes, as well as NK cells. Its activation leads to immunosuppression and functional exhaustion of these cells. Blocking TIM-3 with small molecules can restore T cell effector function and enhance their ability to eliminate tumor cells [330,333].

**Figure 9:**
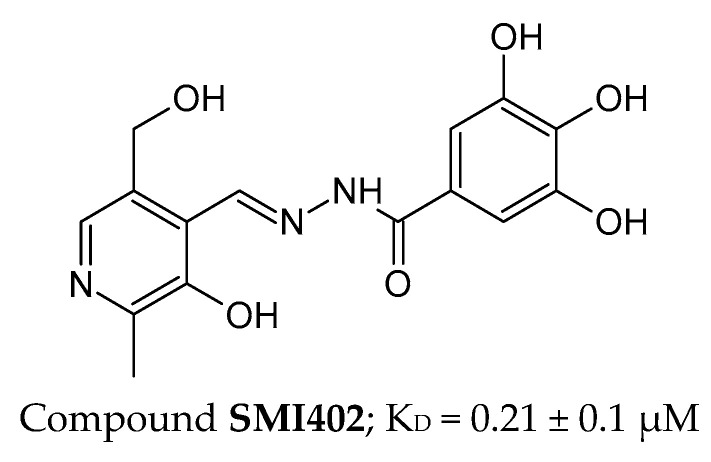
Structure of PD-1 small-molecule inhibitor.

To identify effective TIM-3 inhibitors, a screening study was conducted using virtual molecular modeling and the ChemDiv compound library. Structural analysis of TIM-3 revealed that the most critical ligand interaction site is the FG–CC’ loop, which serves as the central binding region for natural TIM-3 ligands such as phosphatidylserine, CEACAM1, and HMGB1. **SMI402** (Figure 9) emerged as the most promising small-molecule TIM-3 inhibitor among the screened compounds. SMI402 exhibits a high affinity for the ligand-binding site, effectively blocking TIM-3 interactions with its ligands and thereby inhibiting the immunosuppressive signaling pathway [333].

The efficacy of SMI402 has been confirmed both in vitro and in vivo. In in vitro studies, SMI402 effectively inhibited tyrosine phosphorylation in the TIM-3 cytoplasmic tail, indicating its ability to disrupt inhibitory signal transduction. Furthermore, in co-culture assays with CD8+ T lymphocytes, this inhibitor restored the production of proinflammatory cytokines such as IL-2 and enhanced the immune cells’ ability to eliminate tumor cells. Studies in mouse tumor models demonstrated that SMI402 significantly suppresses tumor growth by increasing the infiltration of CD8+ effector T cells and NK cells in the TME. Additionally, SMI402 was particularly effective when combined with PD-1 inhibitors, suggesting that a dual blockade of TIM-3 and PD-1 may provide synergistic therapeutic benefits [333].

The discovery of SMI402 opens new avenues in cancer therapy. Further research should focus on optimizing its chemical structure, improving its stability, and conducting clinical trials to evaluate its efficacy in patients.

#### 6.4.2. Peptides

Peptide inhibitors of TIM-3 represent a promising strategy for blocking the immunosuppressive pathway associated with TIM-3 binding to galectin-9 (Gal-9). Among these, the **peptide P26** (GLIPLTTMHIGK) is of particular significance, as it demonstrates the ability to inhibit the interaction between TIM-3 and Gal-9, potentially reversing T cell exhaustion and enhancing immune responses. The mechanism of P26 action relies on competitive binding to the Gal-9 recognition site on TIM-3, thereby preventing the transmission of the inhibitory signal that usually leads to apoptosis of Th1 helper T cells and the suppression of CD8+ cytotoxic T cell function [334].

The identification of the P26 binding site on TIM-3 was made possible through machine learning approaches and molecular dynamics simulations. Analyses have shown that P26 primarily interacts with the C″–D loop region of the TIM-3 IgV domain, stabilizing through multiple electrostatic and hydrophobic interactions. Notably, its binding induces conformational changes in the protein structure and nearby glycan chains, further disrupting the TIM-3/Gal-9 interaction. This mechanism suggests that P26 may act not only by directly occupying the ligand-binding site but also by inducing structural changes in TIM-3 that negatively impact its ability to interact with Gal-9 [334,335].

Predictive modeling methods for analyzing P26 binding have enabled precise identification of potential interaction sites and optimization of its structure for enhanced TIM-3 inhibition efficiency. Advanced modeling techniques have not only allowed for identifying TIM-3 regions crucial for peptide interaction but also provided insights into the conformational effects induced by peptide binding. As a result, the development strategy for TIM-3 peptide inhibitors can be further optimized for increased stability and effectiveness in blocking the immunosuppressive pathways of TIM-3 [334,335].

## 7. LAG-3

### 7.1. LAG-3 Structure

Lymphocyte-activation gene 3 (LAG-3) is a surface protein belonging to immune receptors, and its expression is particularly high in the TME. Structurally similar to CD4, LAG-3 is found on the surface of activated T cells (CD4+ and CD8+), NK cells, B cells, and plasmacytoid DCs. Its expression increases following antigenic stimulation, and its primary function is to inhibit T cell activation and proliferation, which promotes immunosuppression and can lead to functional T cell exhaustion in tumors [336,337].

LAG-3 consists of four immunoglobulin-like domains (D1-D4), which form a characteristic V-shaped structure. The D1 domain contains the KIEELE motif, which is crucial for the inhibitory function of LAG-3 [338,339,340,341]. The presence of a single lysine residue (Lys468) within the KIEELE motif plays a critical role in transducing inhibitory signals and is essential for suppressing effector T cell functions [342]. LAG-3 has the ability to form homodimers through a conserved hydrophobic region in the D2 domain. Its structure has been resolved at the crystallographic level, enabling precise determination of interactions with ligands, including the major histocompatibility complex class II (MHC-II), fibrinogen-like protein 1 (FGL1), and the TCR–CD3 complex [338,339,340,341].

The interaction of LAG-3 with MHC-II is mediated by loop 2 (residues 103–112) in the D1 domain, allowing for the selective recognition of stable peptide–MHC class II complexes. Additionally, LAG-3 can function independently of MHC-II by interacting with the TCR–CD3 complex within the immunological synapse, leading to the disruption of CD4 and CD8 coreceptor interactions with Lck kinase and a reduction in T cell signaling activation [341].

Detailed structural studies have revealed that LAG-3 undergoes dynamic conformational changes that influence its function and interactions with ligands. Furthermore, LAG-3 can be regulated through proteolytic cleavage by ADAM10 and ADAM17 metalloproteinases, affecting its surface stability and the therapeutic efficacy of its inhibitors. Understanding the structure of LAG-3 is crucial for developing modern immunotherapies, particularly in the context of anticancer mAbs and small-molecule inhibitors targeting key protein domains [338].

### 7.2. LAG-3 Expression in Health and Disease

LAG-3 is expressed in various types of immune cells under both physiological and pathological conditions. In healthy organisms, its expression is tightly regulated and primarily occurs in activated CD4+ and CD8+ T lymphocytes, NK cells, DCs, and subsets of B lymphocytes. LAG-3 expression in T lymphocytes is typically transient and appears in response to antigenic stimulation, suggesting that this receptor functions as a negative regulator that controls excessive immune activation and prevents autoimmunity. Under physiological conditions, LAG-3 helps maintain immune homeostasis, participating in immune tolerance processes and regulating immune responses in mucosal tissues and lymphoid organs [343,344,345,346].

LAG-3 expression is significantly increased, particularly in exhausted T cell populations present in the TME. In cancers such as melanoma, lung cancer, colorectal cancer, and pancreatic cancer, LAG-3 is overexpressed on tumor-infiltrating lymphocytes, contributing to immunosuppression and enabling tumor cells to evade immune attack. LAG-3 overexpression is often correlated with the expression of other inhibitory receptors, such as PD-1 and TIM-3, leading to profound functional exhaustion of T lymphocytes and impairing their ability to eliminate tumor cells. In chronic viral infections, such as HIV and HCV infections, LAG-3 is also upregulated, limiting the effectiveness in immune responses and promoting infection persistence [344,347,348,349,350].

Interestingly, LAG-3 is also involved in autoimmune diseases. In certain conditions, such as rheumatoid arthritis and multiple sclerosis, LAG-3 expression in Tregs may play a role in suppressing excessive immune activity and limiting tissue damage. Conversely, in type 1 diabetes, the absence or insufficient expression of LAG-3 may lead to increased autoimmunity and destruction of pancreatic β cells [345,351,352,353].

One of LAG-3’s key mechanisms of action is its interaction with MHC class II, which leads to blocking the TCR signaling pathway, inhibiting T cell proliferation and increasing the immunosuppressive function of Tregs [2]. In addition to MHC-II, LAG-3 also interacts with other ligands, such as fibrinogen-like protein 1 (FGL1), which inhibits the activity of antigen-specific T cells [352,353]; galectin-3 (Gal-3) [354], involved in T cell anergy in the TME; LSECtin, which affects tumor growth [355]; and the TCR-CD3 complex, which regulates the immune synapse [356]. Studies have shown that LAG-3 is highly expressed in infiltrating T cells in various cancers, including colorectal cancer, NSCLC, pancreatic cancer, and head and neck cancer. Its high expression correlates with immunosuppression and impaired antitumor function of the immune system [338,354,357].

### 7.3. LAG-3 as a Receptor

The LAG-3 acts in multiple ways, modulating the activity of various immune cell types. LAG-3 weakens activation signals through TCR on T lymphocytes by interacting with MHC-II on APCs. This interaction limits T cell proliferation and effector function, promoting exhaustion, particularly in chronic infections and the TME. Moreover, LAG-3 can function independently of MHC-II by interacting with the TCR–CD3 complex within the immune synapse, leading to the dissociation of CD4 and CD8 coreceptors from Lck kinase and further weakening T cell activation [346,350,351,358].

Besides T cells, LAG-3 is also expressed in NK cells, DCs, and B cell subpopulations, where it plays a role in modulating immune functions. In DCs, LAG-3 can influence their antigen-presenting ability and the regulation of T cell responses, whereas in NK cells, it limits their cytotoxicity. In the TME, high LAG-3 expression in immune cells correlates with their exhaustion and a reduced capacity to eliminate tumor cells [337,347,359].

The cytoplasmic region of LAG-3 contains several unique signaling motifs that are not present in other inhibitory receptors, such as PD-1 or CTLA-4. One of the most important is the KIEELE motif, which is crucial for LAG-3’s inhibitory function, although its exact mechanism of action is not yet fully understood. Additionally, the EP motif in the terminal cytoplasmic region is involved in recruiting signaling proteins and modulating immune responses. A unique feature of LAG-3 is its regulation through proteolytic cleavage by ADAM10 and ADAM17 metalloproteinases, which affects its surface stability and the therapeutic efficacy of its inhibitors [354,357,360,361].

LAG-3 is a key inhibitory receptor in the immune system, acting through interactions with MHC-II and other ligands such as FGL1, galectin-3, and the TCR–CD3 complex. Its structure and mechanisms of action make it a significant target for research in modern immunooncology therapies and a potential tool for modulating immune responses in various pathological conditions [341,347,353,362].

### 7.4. LAG-3’s Inhibitors

LAG-3 is a key IC that plays a vital role in immunosuppression and functional exhaustion of T cells, particularly within the TME. Its interaction with MHC class II is dominant in suppressing the immune response, making it an attractive target for modern therapeutic strategies. High expression of LAG-3 in various cancers is associated with impaired antitumor activity of the immune system, and blocking it with mAbs or small-molecular-weight inhibitors shows promise in immunotherapy. With advances in LAG-3 inhibitor research, it is possible to refine further therapeutic strategies that can increase the effectiveness of cancer treatment and improve patient prognosis [347,357,360,363,364].

#### 7.4.1. Small Molecules

Small-molecule inhibitors of LAG-3 represent a new class of compounds developed to block the immunosuppressive effects of this receptor, potentially restoring T cell function and enhancing antitumor responses. The primary mechanism of action of small-molecule LAG-3 inhibitors is to block the interaction between this receptor and its ligand, the major histocompatibility complex class II (MHC-II). LAG-3 has a high affinity for MHC-II, which suppresses T cell activation by limiting their proliferation and effector functions. Small-molecule inhibitors are designed to directly interact with the MHC-II binding site on LAG-3, thereby preventing the formation of an immunosuppressive complex.

**Figure 10:**
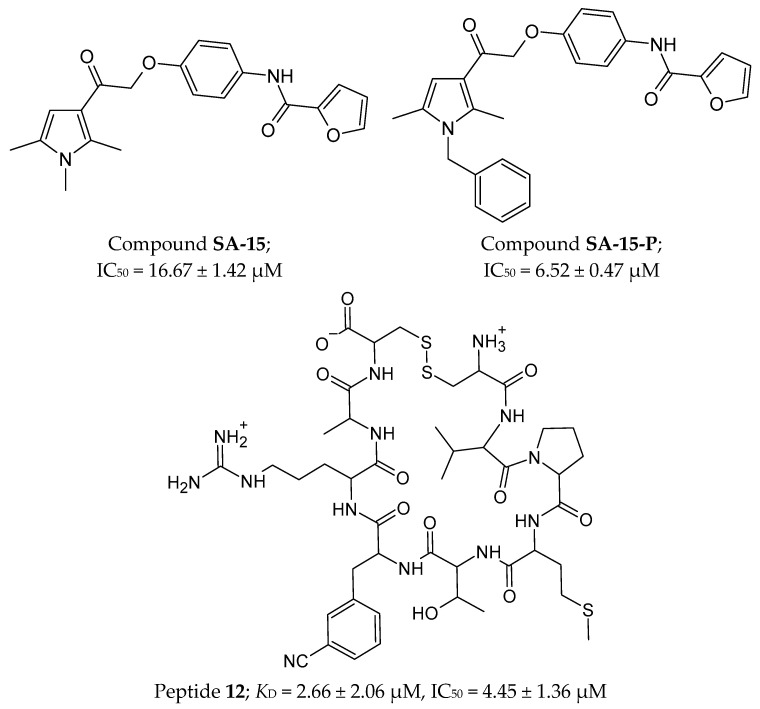
Structure and sequence of LAG-3 peptide and small-molecule inhibitors.

**SA-15** (Figure 10) emerged from a HTS campaign and optimization of the structure–activity relationship (SAR). It is a low-molecular-weight compound designed to inhibit the interaction between LAG-3 and MHC-II. SA-15 demonstrated potent inhibitory activity in biochemical and cell-based assays, effectively restoring T cell function suppressed by LAG-3 engagement. In binding assays, SA-15 inhibited the LAG-3/MHC-II interaction with an IC_50_ of 4.21 μM and also showed an inhibitory effect on the LAG-3/FGL1 interaction with an IC_50_ of 6.52 μM. This dual mechanism of action highlights SA-15’s versatility in blocking multiple immunosuppressive pathways mediated by LAG-3. To improve the pharmacokinetic properties and in vivo efficacy of SA-15, researchers developed **SA-15-P** (Figure 10), a phosphonate prodrug form of SA-15. SA-15-P is more soluble and exhibits improved bioavailability, making it suitable for further preclinical evaluation and potential therapeutic development. Upon administration, SA-15-P is metabolized into its active form (SA-15), which retains full biological activity in disrupting LAG-3-mediated immune inhibition. SA-15-P showed robust activity in cellular assays, enhancing cytokine production (e.g., IFN-γ and IL-2) and restoring the proliferation of previously suppressed T cells [364].

The development of SA-15 and SA-15-P demonstrates a successful approach to translating structural insights into functional small-molecule inhibitors with therapeutic relevance. These compounds not only validate LAG-3 as a druggable target beyond mAbs but also pave the way for future oral immunotherapies with the potential to enhance immune responses in cancer and chronic infections. The promising results achieved with SA-15 and SA-15-P mark an essential milestone in IC modulation using small molecules [364].

#### 7.4.2. Peptides

Peptide inhibitors of LAG-3 are a rapidly developing class of therapeutic molecules that show promising potential in cancer immunotherapy by selectively blocking the interaction of LAG-3 with its ligands. In recent years, two directions of development of these inhibitors have received special attention: linear peptides targeting the LAG-3/FGL1 pathway and cyclic peptides blocking the LAG-3/MHC-II interaction.

The most promising inhibitors of the first type include the **LFP-6** peptide (MHRPPST), which was developed by biopanning from a phage library. LFP-6 selectively inhibits the interaction of LAG-3 with FGL1 (IC_50_ = 11.3 µM) without affecting the classical binding to MHC-II. This is important because FGL1 functions as an MHC-II-independent ligand for LAG-3 activated under conditions of immunosuppression, particularly in tumors. The LFP-6 peptide in its original form contained only L-amino acids, which made it susceptible to rapid proteolytic degradation. In response to this defect, LFP-D1 was developed as a modified version with ends containing D-amino acids, which shows increased biological stability and resistance to degradation under physiological conditions. This peptide retained full blocking activity after 48 h of incubation in serum and tumor cells [363].

LFP-D1 in vitro assays effectively reversed the immunosuppressive effect of FGL1, restoring IL-2 secretion by T cells. In the MC38 animal model, it inhibited tumor growth, increased CD8+ lymphocyte infiltration in the tumor, and stimulated IFN-γ production by CD4+ and CD8+ T cells in lymph nodes and spleen. Moreover, the peptide did not show toxicity or influence on the function of internal organs. Based on LFP-D1, a bispecific peptide LFOP was also developed, which combines the ability to block LAG-3/FGL1 and PD-1/PD-L1. LFOP showed more potent T cell activation and proliferation than any monotherapies and a synergistic effect in combination with radiotherapy [363].

The second significant trend in the development of peptide inhibitors of LAG-3 is cyclic peptides, which offer higher stability, bioavailability, and affinity to the molecular target due to the closed conformation and disulfide bridges. In a study on cyclic LAG-3 inhibitors, a new series of peptides based on the cyclic structure (Cys-Val-Pro-Met-Thr-Tyr-Arg-Ala-Cys) was developed, among which the best profile was demonstrated by **peptide 12** (Figure 10). In this analog, the tyrosine residue was substituted for L-3-cyanophenylalanine, significantly improving its inhibitory activity (IC_50_ = 4.45 µM) and affinity to LAG-3 (K_D_ = 2.66 µM). Simulation studies have shown that the cyano substituent enhances π–π interactions and creates additional hydrogen bonds in the binding site, promoting stable peptide conformation and its better alignment with LAG-3/MHC-II. The cyclic structure of peptide 12 provides higher resistance to enzymatic degradation and maintains an active conformation in serum for at least 24 h. However, no apparent therapeutic effect has been observed in animal models, which may be due to the non-specific binding of the peptide to serum proteins and its limited bioavailability at the site of action [365].

Peptide LAG-3 inhibitors—both selective inhibitors of the LAG-3/FGL1 pathway, such as LFP-6 and LFP-D1, and cyclic LAG-3/MHC-II inhibitors, such as peptide 12—represent two complementary approaches to modulate LAG-3 function in cancer immunotherapy. Both classes demonstrate high affinity and stability, and further studies on their pharmacokinetics, bioavailability, and combination therapies (e.g., radiotherapy or PD-1 inhibitors) may significantly contribute to developing effective next-generation drugs [359,363,365].

## 8. TIGIT

### 8.1. TIGIT Structure

TIGIT (WUCAM [366], Vstm3 [367], or VSIG9 [368])—a T cell immunoreceptor with immunoglobulin and an ITIM domain—is another receptor that serves as a negative regulator of T cell functions [369]. This membrane protein consists of 244 amino acids [370] and is a member of the CD28 family [367]. Its structure includes an immunoglobulin variable domain, a type 1 transmembrane domain, and a cytoplasmic tail with two inhibitory motifs: ITIM and an Ig tail tyrosine (ITT)-like motif, which activates the main inhibitory signals that suppress T cell activation [115,370,371].

The IgV domain is structured with two layers of six (A′GFCC′C″) and four (DEBA) strands in β-sandwiches [372,373]. It contains several exposed hydrophobic residues (Leu65, Ile68, Leu73, Phe107, Ile109, Pro124, and Phe123), which form the surface interacting with ligands [373]. Additionally, in the FG loop, a TYP motif with a conserved aromatic residue, Tyr113, interacts with the hydrophobic pocket of ligands [372]. TIGIT exists as a homodimer, with the core of the interaction surface being the Ile42 residue located in the extracellular region, which binds to a groove formed by the residues Thr29 and Cys45 [372]. Further stabilization is provided by interactions between the two β-strands A’, which interact in an antiparallel manner to create a dense network of hydrogen bonds [372].

As a result of TIGIT interaction with its ligands, the phosphorylation of Tyr225 within the ITT-like motif occurs, triggering the main inhibitory signaling pathways [115,370,371], which limit, among other things, NK cell activation [374]. Mutation of this critical residue leads to a complete loss of TIGIT activity [375]. The cytosolic adapters Grb2 and β-arrestin bind to the ITT-like motif, engaging SH2-containing inositol phosphatase-1 (SHIP-1), which disrupts the activation of TRAF6 and NF-κB, thereby inhibiting the secretion of IFN-γ by NK cells [376]. Similar to PD-1, CTLA-4, and BTLA, TIGIT can recruit SHP2 via the ITIM motif and phosphorylation of Tyr231 [37,376].

### 8.2. TIGIT Expression in Health and Disease

TIGIT is an important IC in modulating innate and adaptive immune responses [376]. Its inhibitory action affects various cell types, such as T cells, NK cells, Tregs, and follicular T helper cells [119,131,371]. Notably, TIGIT is not present on naive T cells [216,366] but appears on activated T cells expressing PD-1, indicating their advanced activation [131,140]. This receptor is also found on the surface of memory B cells, which control the immune response by blocking the proinflammatory functions of DCs and T cells [347,377]. Importantly, TIGIT overexpression in NK cells (compared to other ICs like PD-1) leads to an apparent inhibition of their functionality, which has implications for immunotherapy [378,379]. Studies have also shown that the level of TIGIT is elevated in both the peripheral blood cells of healthy individuals and cancer patients, suggesting its role in suppressing immune responses in the context of cancer [371].

TIGIT, alongside BTLA, is an inhibitory receptor that regulates innate and adaptive immunity through various mechanisms [347,371,378]. Primarily, the binding of TIGIT to its ligands triggers the activation of signaling pathways that influence the immune response [380]. In addition to the ITIM motif, which recruits SHP tyrosine phosphatase proteins and inhibits INF-γ secretion, TIGIT also exhibits functions similar to the CTLA-4/CD28 system, with CD226 acting as the competing receptor for TIGIT [121,216,377,380]. CD226 is expressed more extensively than TIGIT and is also present on B cells, monocytes, and platelets [376]. TIGIT interferes with the homodimerization of CD226, hindering its interaction with shared ligands CD155 and CD112, thereby disrupting T cell co-stimulation [213,347,380]. Furthermore, TIGIT has a higher affinity for CD155 than CD226, inhibiting CD226 activation pathways and suppressing the immune response [376]. Increased secretion of IL-10 and reduced secretion of IL-2 and IL-12 by DC and macrophages expressing CD155 hampers both innate and adaptive immunity, resulting in enhanced immunosuppressive effects due to disrupted proliferation, function, and activation of T and NK cells [119,131,371,378]. CD226 activity can be restored by blocking PD-1 and TIGIT receptors [376]. The interaction of TIGIT/CD155 promotes immune tolerance toward DCs [347].

Most disorders associated with TIGIT arise from its deficiency or excessive expression in various cell types, leading to alterations in signaling pathways [381]. For instance, the absence of TIGIT on T cells does not directly induce autoimmunization; however, the blockade of signaling pathways triggered by TIGIT interactions with its receptors exacerbates several autoimmune conditions [216,382]. The critical role of TIGIT in maintaining internal organ homeostasis is further corroborated by its reduced expression on T cells in kidney dysfunction [383]. Due to elevated expression levels in T cells, TIGIT may serve as a clinical biomarker in systemic lupus erythematosus (SLE) [382] and rheumatoid arthritis [132,384]. Moreover, inhibitors targeting the TIGIT/CD155 interaction between lymphoid cells and macrophages may enhance lymphoid cell survival, thus showing therapeutic potential in chronic allergic conditions [382].

A reduction in TIGIT expression in memory B cells has been linked to central nervous system inflammation and subsequent multiple sclerosis development [385]. A notable decrease in TIGIT levels in CD4+ T cells has also been observed in patients with psoriasis [386]. Emerging research suggests that the gut microbiome regulates innate immune responses in a manner dependent on TIGIT expression [371]. In gastrointestinal disorders such as ulcerative colitis and Crohn’s disease, TIGIT levels in CD38+ effector T cells were lower than healthy controls [382].

Furthermore, in the context of partial PD-1 signaling impairment in type 1 diabetes, TIGIT is crucial in preserving the partial functionality of infiltrating T cells [387], particularly in the presence of anti-PD-1 antibodies [130]. In HIV-infected patients, a significant correlation has been observed between disease progression and the concurrent expression of TIGIT and PD-1 [121]. Interestingly, elevated expression of ICs in HIV-infected cells appears to inhibit viral transcription, thereby promoting HIV latency [388].

The expression of TIGIT exhibits variability across different cell types and within cellular locations, which may result in ambiguous assessments of TIGIT expression within the TME [370]. TIGIT and the accumulation of regulatory T cells in tumor tissues are primarily associated with tumor progression and reduced effector functions against malignant cells, ultimately leading to immune escape of tumor cells [347]. TIGIT may be co-expressed in tumor cells alongside other receptors, including PD-1, TIM-3, and LAG-3 [371]. Overexpression of TIGIT has been observed in patients with hepatocellular carcinoma associated with HBV infection [124], pancreatic adenocarcinoma [389], NSCLC, and colorectal carcinoma [216]. Increased TIGIT expression on CD8+ T cells has been linked to poor prognosis in patients with relapsed leukemia and those with advanced colorectal cancer [347]. Elevated TIGIT expression has also been reported in ovarian cancer, where it enhances the immunosuppressive activity of Tregs, thereby impacting patient mortality [376]. Additionally, the absence of TIGIT expression in NK cells has been shown to delay tumor growth and improve survival [347].

### 8.3. TIGIT as a Receptor

The ligands interacting with TIGIT include CD155 (poliovirus receptor—PVR [369,390], Necl5 [391], Tage4 [392]), CD112 (nectin-2 [393], PVRL2 [394]), CD113 (nectin-3, PVRL3 [395]), nectin-4 (PVRL4) [396,397], and Fab2 [7,398]. The most well studied in terms of interaction with TIGIT are CD155 and CD112. These molecules are similar to nectins, belonging to the receptor family that plays a critical role in the adhesion, migration, proliferation, morphogenesis, and differentiation of various cell types [375,381,399,400]. Both ligands are expressed by both APCs and tumor cells [131,366,373], as well as by T cells and non-hematopoietic cells [379,401]. Their presence has also been detected on fibroblasts and endothelial cells [216]. Both ligands exhibit a higher affinity for TIGIT than CD226 [373,402], with CD155 having a higher affinity for TIGIT than CD112 [131]. CD155 also binds to the CD96 receptor, while CD112 interacts with the CD112R receptor [376].

In complexes with the TIGIT homodimer, both ligands form a heterotetrameric structure through a “lock-and-key”-type interaction, with two TIGIT molecules at the center surrounded by single molecules of CD112 or CD155 [372,373,374]. The interaction with TIGIT occurs on both proteins’ front β-sheets (A′GFCC′C″) surfaces, with the C′C″ loops interacting with the FG loops [372,399]. Both ligands contain three unique and highly conserved motifs in the sequence of the first immunoglobulin variable IgV-like domain: the VSQ, AX6G, and TFP motifs, located on strands C, C′, and F [369,372,374]. In the FG loop, CD155 contains the TFP motif, which includes a conserved aromatic residue—F128, fitting into a hydrophobic pocket in the C′C″ loop of the TIGIT receptor [372,399]. Mutations within these motifs result in weakened or absent TIGIT/CD155 interaction [372]. Similarly, mutations in the AX6G region of CD112 reduce its affinity for TIGIT and decrease the thermostability of the ligand itself [374]. The TIGIT/CD155 interaction induces tyrosine phosphorylation in the ITT domain. It recruits the phosphatase SHIP1, leading to the inhibition of the PI3K, MAPK, and NF-κB signaling pathways, as well as the production of proinflammatory cytokines, such as IL-12, in DCs [216].

Increased expression of both ligands has been confirmed in various cancers. Overexpression of CD155 has been linked to poor prognosis in cancers such as colorectal cancer, NSCLC [390], melanoma [391], pancreatic cancer [370,389], and cervical cancer [376]. CD112 is strongly expressed in bone marrow, kidneys, pancreas, spleen [403], and lung cells, as well as in breast, ovarian [400], and prostate [394] cancers. Its overexpression in some cancer types may be attributed to genotoxic stress [216].

### 8.4. TIGIT’s Inhibitors

TIGIT is also a promising, novel molecular target in cancer immunotherapy, which is also due to its synergistic action with other ICIs [404]. Currently, anti-TIGIT antibodies, Tiragolumab and Ociperlimab, are in Phase III clinical trials [370,390,399,405]. In combination therapy, together with anti-PD-1 antibodies and conventional chemotherapy, these antibodies demonstrate activity in the treatment of NSCLC, small-cell lung cancer (SCLC), squamous cell carcinoma of the esophagus, hematological malignancies, and melanoma [370,390,405]. Notably, the affinity of Ociperlimab for TIGIT increases as the pH drops from 7.4 to 6.0, suggesting that the interaction between TIGIT and its ligands may be pH-dependent [399].

#### 8.4.1. Small Molecules

**Figure 11:**
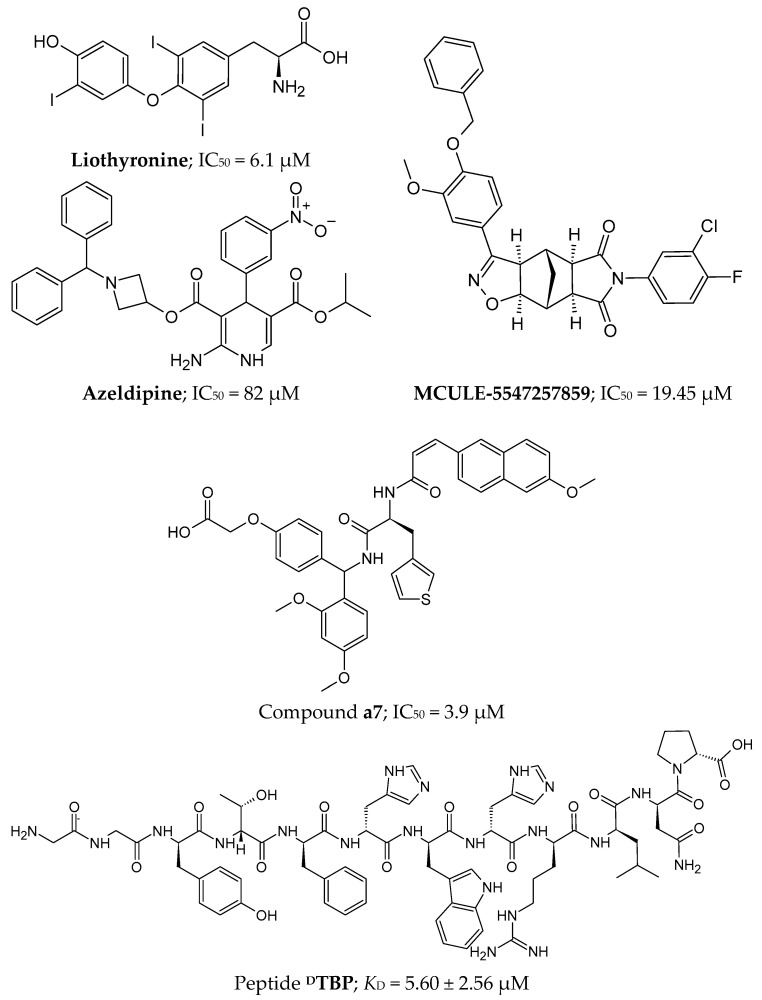
Structures of TIGIT small-molecule and peptide inhibitors.

The inhibitors of the TIGIT/CD155 interaction described so far were identified based on the crystal structure analysis of the TIGIT/CD155 complex, followed by docking and optimization of small molecule structures using the MOE software [406,407]. Several molecules that bind to CD155 and block the TIGIT/CD155 interaction were proposed, resulting in increased numbers and functionality of T cells [406,407]. **Liothyronine** (IC_50_ = 6.1 μM, Figure 11), an artificial form of the thyroid hormone triiodothyronine, was one of these inhibitors [407]. Similarly, **azelnidipine** (Figure 11), a registered drug used as a calcium channel blocker [408], also has potential as a TIGIT/CD155 interaction inhibitor (IC_50_ = 82 μM) [406]. A glutamine derivative, **Gln(TrT)**, also binds to CD155 (K_D_ = 2.95 ± 1.8 μM) but also tends to bind to PD-1 (K_D_ = 4.4 ± 3.8 μM) [371]. Other examples of inhibitors include **hemine** (IC_50_ = 19.86 ± 1.24 μM) and **protoporphyrin IX** (IC_50_ = 21.69 ± 2.23 μM), which induce ferroptosis in cancer cells and restore IL-2 secretion from Jurkat cells [409].

Another approach involved screening a library of compounds using machine learning and centroid-based docking methods, which led to the identification of 14 potential small-molecule inhibitors of the TIGIT/CD155 interaction, including the previously studied hemine and liothyronine inhibitors [410]. The best parameters were found for **compound 1**, **MCULE-5547257859** (Dock score = −7.8 kcal/mol; IC_50_ = 19.45 μM; see Figure 11) [410]. Using a DNA-encoded library (DEL) platform combined with retrained machine learning models, a hit compound comprising three distinct building blocks was identified, exhibiting an IC_50_ value of 20.7 µM against the TIGIT receptor [411]. Subsequent structure-based optimization led to the design of a focused series of analogs. Among these, **compound A7** (Figure 11) demonstrated the highest potency, with an IC_50_ value of 3.9 µM, representing a significant improvement over the initial hit [411]. However, virtual docking should be transferred to laboratory settings to verify the inhibitor’s effect on the TIGIT/CD155 interaction and its impact on cells.

#### 8.4.2. Peptides

Using the mirror-image phage display technique, the only peptide identified to date as an inhibitor of the TIGIT/CD155 interaction is ^D^TBP-3 (Figure 11) [412]. This molecule exhibited an affinity in the range of 5.60 μM, and its efficacy was demonstrated in vivo in colorectal cancer and melanoma models [412]. The ^D^TBP-3 peptide interacts with TIGIT at the same structural site as CD155 [179].

## 9. Future Perspective—CD112R

The immune system relies on a complex network of stimulatory and inhibitory receptors to precisely regulate the activation of effector cells, such as T cells and NK cells. This review has focused on ICs for which inhibitors based on small molecules, peptides, and PROTACs are currently under development. However, other inhibitory receptors should not be overlooked, as they may represent future therapeutic targets in cancer and autoimmune diseases. Among them, CD112R (also known as PVRIG), a recently identified member of the PVR/nectin family, is gaining increasing attention.

CD112R shares structural features with other members of the PVR family, comprising three domains: a single extracellular IgV-like domain, a transmembrane region, and a long cytoplasmic tail containing an ITIM-like motif. It also harbors conserved sequence motifs such as Tyr139, AX6G, and VSQ, which are typical of this receptor class [413,414]. CD112R is expressed on the surface of T and NK cells, and its expression is regulated by co-stimulatory and coinhibitory signaling networks involving TIGIT, CD96, and CD226 [401,415]. The only known human ligand for CD112R is CD112, which binds CD226 and TIGIT with significantly lower affinity. Due to its much higher binding affinity to CD112, CD112R can outcompete CD226, promoting suppression of T cell proliferation and effector function within TME [401,413,414,415].

Functional studies have demonstrated that the blockade of CD112R—particularly in combination with TIGIT inhibitors—leads to enhanced cytokine production, including IFN-γ, IL-2, IL-5, IL-10, and IL-13, and increases the cytotoxicity and expansion of CD8+ T cells [413]. Although the precise mechanisms underlying CD112R/CD112 signaling remain incompletely understood, the available data suggest a promising antitumor potential [416]. In preclinical tumor models, treatment with anti-CD112R mAb alone or in combination with PD-1 blockade led to improved antitumor immune responses and inhibited tumor growth, including in models of colorectal cancer [417].

In studies of NSCLC, combination therapy with anti-CD112R and anti-TIGIT antibodies resulted in significantly higher levels of IL-2 and IFN-γ compared to monotherapy with either agent [418]. Furthermore, CD112R blockade enhanced NK cell cytotoxicity and suppressed tumor progression in breast cancer models through increased IFN-γ production [419]. Significantly, the CD112 to CD155 expression ratio varies across tumor types, which can influence the immunosuppressive balance within the CD112R/CD112 and TIGIT/CD155 axes [401].

Currently, the anti-CD112R antibody COM701 is being evaluated in early-phase clinical trials (NCT03667716) in patients with advanced solid tumors, including breast, ovarian, endometrial, and non-small-cell lung cancers. These trials aim to explore the safety, pharmacodynamics, and therapeutic efficacy of CD112R inhibition in combination with other ICTs [418].

## 10. Conclusions

Peptide and small-molecule inhibitors of negative ICs represent one of the most promising avenues in the development of modern immunotherapy. Their significance stems primarily from the limitations associated with conventional mAbs, such as pembrolizumab, nivolumab, and ipilimumab. Although these antibodies have revolutionized the treatment of many cancers, their sizeable molecular size poses several disadvantages—limited tissue penetration, prolonged half-life, challenges with oral administration, high immunogenicity, and the potential for autoimmune-related complications. As a result, there is an ongoing search for alternative approaches capable of effectively and selectively modulating the immune response while minimizing adverse effects.

Peptides and small molecules offer numerous advantages over antibodies. Due to their smaller size, these inhibitors can more readily penetrate the TME, are eliminated more rapidly, are easier to modify chemically, and can often be administered orally. Furthermore, they exhibit lower immunogenicity, significantly reducing the risk of therapy-associated complications.

The discussed strategies encompass a comprehensive overview of key negative ICs, including CTLA-4, PD-1, VISTA, TIM-3, BTLA, LAG-3, and TIGIT. Peptide and small-molecule inhibitors targeting these checkpoints are pivotal in advancing next-generation immunotherapies. Their development helps overcome many of the challenges posed by mAb therapies, offering more effective, selective, less toxic, and more easily administered therapeutic alternatives. With the expanding understanding of the molecular mechanisms governing immune regulation and the ability to precisely design chemical structures, this class of inhibitors holds significant potential to become a cornerstone of future therapeutic strategies, including oncology and autoimmune diseases.
